# Bitbow Enables Highly Efficient Neuronal Lineage Tracing and Morphology Reconstruction in Single *Drosophila* Brains

**DOI:** 10.3389/fncir.2021.732183

**Published:** 2021-10-20

**Authors:** Ye Li, Logan A. Walker, Yimeng Zhao, Erica M. Edwards, Nigel S. Michki, Hon Pong Jimmy Cheng, Marya Ghazzi, Tiffany Y. Chen, Maggie Chen, Douglas H. Roossien, Dawen Cai

**Affiliations:** ^1^Department of Cell and Developmental Biology, University of Michigan, Ann Arbor, MI, United States; ^2^Biophysics LS&A, University of Michigan, Ann Arbor, MI, United States; ^3^Neuroscience Graduate Program, University of Michigan, Ann Arbor, MI, United States

**Keywords:** multicolor transgenics, lineage tracing, morphological analysis, Bitbow, Brainbow, *Drosophila* brain

## Abstract

Identifying the cellular origins and mapping the dendritic and axonal arbors of neurons have been century old quests to understand the heterogeneity among these brain cells. Current Brainbow based transgenic animals take the advantage of multispectral labeling to differentiate neighboring cells or lineages, however, their applications are limited by the color capacity. To improve the analysis throughput, we designed Bitbow, a digital format of Brainbow which exponentially expands the color palette to provide tens of thousands of spectrally resolved unique labels. We generated transgenic Bitbow *Drosophila* lines, established statistical tools, and streamlined sample preparation, image processing, and data analysis pipelines to conveniently mapping neural lineages, studying neuronal morphology and revealing neural network patterns with unprecedented speed, scale, and resolution.

## Introduction

Bilaterian nervous systems are built upon heterogeneous populations of neurons that form interconnected circuits. To understand the molecular and cellular mechanisms that lead to proper circuit formation, it is critical to elucidate the lineage origin and morphogenesis of neurons. This is because lineages mark the outcome of neurogenesis, while morphology dictates the circuit structure by defining physical boundaries of the receptive and projective fields. Tremendous efforts have been made in the past century to take on these two fundamental quests in neuroscience, evolving from methodologies that can cope with one or a few neurons at a time, such as stochastic silver staining (Golgi’s method) (Golgi, 1885; Ramon y Cajal, 1911) and mosaic genetic labeling (Lee and Luo, 1999; Muzumdar et al., 2007), to multispectral labeling technologies (Brainbow) that can differentiate large population of neurons in the same tissue (Livet et al., 2007).

Brainbow and Brainbow-like tools label neurons in distinct colors by expressing random ratios of different fluorophores, such as fluorescent proteins (FPs), upon genome recombination (Lichtman et al., 2008; Richier and Salecker, 2015; Weissman and Pan, 2015). Reagents, including mice (Livet et al., 2007; Snippert et al., 2010; Cai et al., 2013), fruit flies (Hadjieconomou et al., 2011; Hampel et al., 2011; Förster and Luschnig, 2012; Boulina et al., 2013; Worley et al., 2013; Chin et al., 2014; Kanca et al., 2014), zebrafish (Pan et al., 2011; Gupta and Poss, 2012; Pan et al., 2013; Robles et al., 2013), bacteria (Barbier and Damron, 2016), and viruses (Kobiler et al., 2010; Cai et al., 2013; Chan et al., 2017; Sakaguchi et al., 2018) are now broadly available for lineage and morphology studies. In lineage studies, unique colors generated in the progenitor cells and inherited by their progenies were used to depict the clonal expansion process of adjacent lineages (Snippert et al., 2010; Gupta and Poss, 2012; Worley et al., 2013; García-Moreno et al., 2014; Loulier et al., 2014). In morphology studies, the unique colors of neurites aided in identification of parallel projection patterns (Robles et al., 2013; Nern et al., 2015) and confirming presynaptic inputs from multiple neurons converging to a common target (Hammer et al., 2015; Takesian et al., 2018; Roossien et al., 2019; Shen et al., 2020). However, current designs are often limited to generating up to tens of reliably distinguishable colors in a transgenic animal. The small unique color pool results in a high probability of labeling neighboring cells with the same color, therefore constraining the labeling density for neuronal morphology reconstructions. This makes it even more challenging to interpret lineage tracing results due to the need for unique colors to specify cells in the same lineage. In addition, distinguishing color variants differing by intensity levels in spectral channels is not reliable for lineage tracing because FP expression level may vary among cells in the same lineage.

One way to generate more unique labels for lineage tracing is to localize the same FPs to different subcellular compartments. In strategies such as CLoNe and MAGIC, Brainbow cassettes targeted to cytoplasm, cell membrane, nucleus, and/or mitochondria were co-electroporated with transposase for genome integration, which allowed the differentiation of neighboring progenies in chick and mouse embryos with fewer color collisions (García-Moreno et al., 2014; Loulier et al., 2014). However, the number of expression cassettes being integrated in each cell is random in these experiments, leading to uncertainty in each color’s appearance probability which complicates quantitative analysis. The Raeppli strategy solves this problem by generating a transgenic *Drosophila* which utilizes 4 FPs to create up to 4 × 4 = 16 membrane and nucleus color combinations (Kanca et al., 2014). In parallel, strategies such as TIE-DYE and MultiColor FlpOut (MCFO) attempt to generate more color combinations by stochastically removing the expression stops from each FP module (Worley et al., 2013; Nern et al., 2015). While inserting 3 different modules into 3 genomic loci allows generating up to 2^3^−1 = 7 unique labels, it is difficult to insert more modules to more genomic loci in a single transgenic animal.

Here we present Bitbow, a digital format of Brainbow to greatly expand the unique color pool from a single transgenic cassette. Unlike the original Brainbow, whose FP choices are exclusive in one cassette, Bitbow allows each FP to independently express in an ON or OFF state upon recombination. Color coding by each FP’s binary status is similar to the information coding by each bit in computer memory, thus leading to the name Bitbow. In a recent study, we implemented the Bitbow1 design to target 5 spectrally distinct FPs to the nucleus for lineage tracing (Veling et al., 2019). Here, we present novel Bitbow1 flies which encode up to 32,767 unique “colors” (Bitbow codes) in a single transgenic animal. This allows reliable lineage tracing without complicated statistical tests (Veling et al., 2019). To better enable morphology tracing, we generated Bitbow2, which couples Bitbow1 to a self-regulating recombination mechanism. This enables generating consistent neuronal labeling by a simple cross of a Bitbow2 fly to an enhancer-Gal4 driver fly without the need for heat-shock.

## Results

### Characterization of Bitbow Design in the *Drosophila* Brain

**FIGURE 1:**
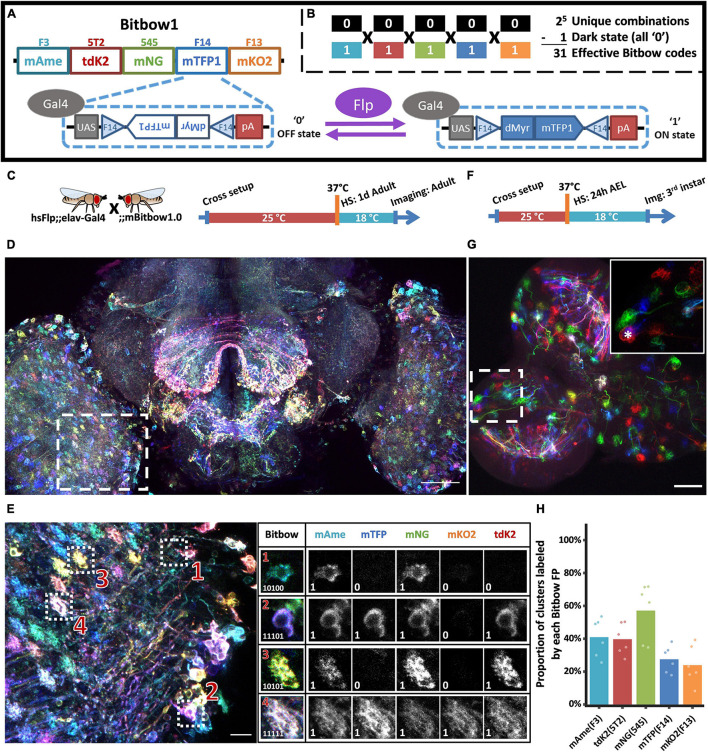
Bitbow1 design and characterization of labeling properties. **(A)** Schematic of Bitbow1 design. Five spectrally distinct FPs are separated by five pairs of reversely positioned orthogonal FRT sites. The mTFP/FRT-F14 module is shown in the dashed box. The FP’s open reading frame (ORF) is positioned in the reverse direction, corresponding to a default OFF state (‘0’). Upon Flp induced recombination, the FP’s ORF may spin to the forward direction for Gal4 driven expression, corresponding to an ON state (‘1’). **(B)** 31 Bitbow color codes could be generated in a single Bitbow1 brain. **(C)** A hsFlp;elav-Gal4 driver fly was crossed to the mBitbow1.0 fly to examine the offspring expression in the nervous system upon heat-shock induced Flp activity. Experimental setups of adult heat shock-induced labeling. **(D)** Maximum intensity projection overview of an adult heat-chocked brain. **(E)** Left panel, enlarged boxed region in panel **(D)** showed individual neurons are labeled in distinct colors, i.e., Bitbow codes. Right panel, Bitbow codes of four selected optic lobe neurons’ somas or terminals. **(F)** Experimental setups of generating heat shock-induced Bitbow labeling in 3rd instar brains. **(G)** Maximum intensity projection overview of a 3rd instar larvae heat-chocked brain. Inset, the enlarged boxed region showed clusters of cells labeled in the same colors. Asterisk indicates a neuroblast. **(H)** Quantification of occurrence frequencies of each Bitbow color. Among all quantified clusters, the fraction of clusters containing each Bitbow color were displayed. 787 clusters from 6 brains are included. Each dot on the graph represents quantification from one brain. Scale bars: **(D,G)** 50 μm, **(E)** 10 μm.

To permit independent recombination of each FP, we utilized a pair of inverted FRT sites flanking a reversely positioned FP, which is downstream of a 10xUAS sequence and upstream of a polyadenylation sequence (Figure 1A). This default OFF state guarantees a non-fluorescent expression. Upon Flp recombination, the flanked FP spins between the inverted FRT sites, resulting in either an ON or OFF state of expression driven by Gal4. Such a design exponentially increases the color-coding capacity with increasing numbers of bits (FPs) in the same transgenic animal (Figure 1B), however, requires a transient Flp activity to ensure the recombination choice is stabilized, similar to the original Brainbow2 design (Livet et al., 2007). In order to guarantee independent recombination between each FP, we used incompatible flanking FRT sequences. Other than the three previously used incompatible Frt sites (Cai et al., 2013), FRT-F3, FRT-5T2, and FRT-545, we identified FRT-F13, FRT-F14, and FRT-F15 as additional incompatible sites in a screen (Supplementary Figure 1; Turan et al., 2010). As FRT-F15 has lower recombination efficiency (data not shown), we ended up with a 5-bit Bitbow1.0 design that consists the other five FRT sites to control the independent recombination choices of mAmetrine, mTFP1, mNeonGreen, mKusabira-Orange2 and tdKatushaka2, respectively (Ai et al., 2006, 2008; Sakaue-Sawano et al., 2008; Shcherbo et al., 2009; Shaner et al., 2013). These FPs were chosen for their brightness, photo-stability, antigenicity, and spectral separation (Supplementary Figure 2). Finally, we generated a cell membrane-targeting Bitbow1.0 (mBitbow1.0) fly to better reveal whole neuron morphology. A 10 amino acid myristoylation signal peptide from *Drosophila* dSrc64B was used to target the FPs onto the cell membrane (Struhl and Adachi, 1998).

Next, we crossed mBitbow1.0 flies to hsFlp;elav-Gal4 driver flies to examine the offspring expression in the nervous system upon heat-shock induced transient Flp activity. When young adult offspring were heat-shocked at 1 day after eclosion and imaged at 3 days later (Figure 1C), we observed individual neurons expressing unique combinations of Bitbow codes (Figure 1D). We found that after spectral unmixing (detailed in the section “Materials and Methods”), the normalized fluorescence intensity in each positively expressed cell can form a clear population that was well separated from the intensity coming from residue fluorescence or background noise (Supplementary Figure 3). Therefore it is easy and reliable to denote each cell’s Bitbow code as a series of 5-bit 0/1 digits (Figure 1E). Increasing the number of heat-shocks (thus Flp activity) increased the total number of neurons being labeled from tens to hundreds in a single optical lobe (Supplementary Figures 4A–C). The median number of FP species being expressed in the same neuron also increases from two to three (Supplementary Figure 4D). Nonetheless, all 31 expected Bitbow codes were identified regardless of the number of heat shocks, albeit each of which was observed with a different frequency (Supplementary Figure 5). The appearance of strong and diverse Bitbow code labeling days after transient heat-shock also indicated that recombination outcomes induced by transient Flp activity were stable. Otherwise, all FPs would keep spinning so that they would all have some transcripts positioned in the forward direction to become fluorescent in all cells. Because the uneven recombination frequency of each FP will result in reduced lineage coding ability, we calculated the Shannon entropies of the Bitbow codes generated from different heat shock experiments as an estimation of their information carrying ability. We found that the Shannon entropies of the mBitbow1.0 flies were 3.67, 3.97, or 4.03 for the 1-, 2-, or 3-heat shock experiments, respectively, indicating that multiple heat shocks increase the labeling coverage as well as increase Bitbow’s information-carrying capacity.

Depending on the timing of heat-shock, stochastic colors can be observed in neighboring neurons or clusters of neuronal progenies if recombination happens in postmitotic neurons or progenitor cells, respectively (Loulier et al., 2014). While post-eclosion heat-shock demonstrated the former situation, the later situation can be examined by heat-shocking at 24 h after egg laid (24 h AEL, i.e., early 1st instar larval stage) and imaging at 72 h post heat-shock, at the 3rd instar larval stage (Figure 1F). Interestingly, while there are plenty of postmitotic neurons at the 1st instar larval stage, most neighboring neurons were labeled as cell clusters in the same Bitbow code (Figure 1G). In addition, we always observed a much larger size neuroblast (NB, i.e., neural stem cell) being labeled in the same Bitbow code in each cluster (Figure 1G inset, asterisk). Collectively, these observations suggested that under the heat-shock conditions optimized for larvae survival, recombination events mostly happened in the NBs and the recombination outcome did not change over time. Quantification of the expression frequency of each FP, i.e., the recombination rate of each FRT site, indicates that FRT-545 has the highest recombination rate, followed by FRT-F3, and FRT-5T2, while FRT-F14 and FRT-F13 have the similarly lowest among the five (Figure 1H). This observation is not specific to the membrane targeting, but is consistent in other Bitbow1.0 flies (detailed below).

### Targeting Bitbow Fluorescent Proteins to Multiple Subcellular Compartments Permits High-Throughput Lineage Tracing in the Whole *Drosophila* Brain Without Ambiguity

**FIGURE 2:**
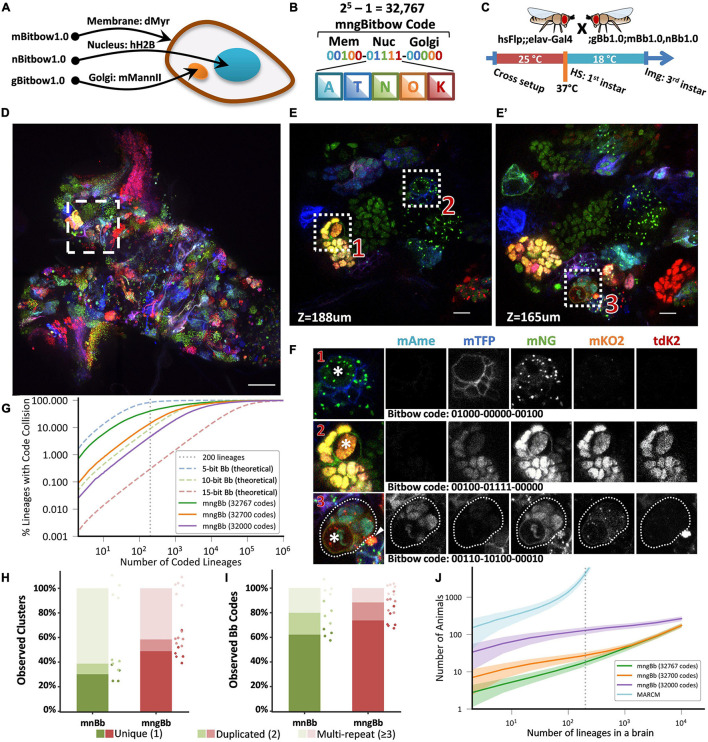
Targeting Bitbow fluorescent proteins (FPs) to multiple subcellular compartments enables high-throughput lineage tracing without ambiguity. **(A)** The same Bitbow FPs are targeted to cell membrane, nucleus, or Golgi apparatus to generate spectrally-spatially resolvable Bitbow codes. **(B)** Up to 32,767 unique mngBitbow codes can be generated can be presented as 3 groups of 5-digit 0/1s in correspondence with the expression status of mAmetrine (A), mTFP1 (T), mNeonGreen (G), mKO2 (O) and tdKatushka2 (K). **(C)** Experimental setup of generating heat-shock induced mngBitbow1.0 labeling and imaging. **(D)** Maximum intensity projection of a 3rd instar mngBitbow1.0 brain. Scale bar, 50 μm. **(E,E’)** Two confocal image slices corresponding to two different z positions in the boxed region in panel **(B)**. Scale bar, 10 μm. **(F)** 3 clusters marked in panels **(E,E’)** are assigned mngBitbow barcodes. Asterisks indicate the neuroblasts of each cluster. The arrowhead highlights an adjacent neuroblast labeled by a distinct mngBitbow code. **(G)** Simulation of Bitbow code collision in lineage mapping experiments. Dashed curve lines are simulations based on theoretical Bitbow code frequencies. Solid curve lines are simulations based on observed Bitbow code frequencies. Vertical dotted line corresponds to mapping all of the 200 lineages in a single adult *Drosophila* central brain. **(H)** Percentages of cell clusters that are uniquely labeled, or 2 of them, or ≥3 of them are labeled by the same mnBitbow (286 clusters, 4 brains) or mngBitbow (577 clusters, 6 brains) codes in each brain. Means and all data points are shown. Each dot represents quantification from one brain, and dots are colored in the same way as the stacked bar graphs. **(I)** Percentages of mnBitbow (*N* = 80, 4 brains) or mngBitbow (*N* = 240, 6 brains) codes that are expressed in 1, or 2, or ≥3 clusters in each brain. Means and all data points are shown. Each dot represents quantification from one brain, and dots are colored in the same way as the stacked bar graphs. **(J)** Monte Carlo simulations estimate the number of animals that are needed (*y*-axis) to sample all lineages at least once in animal brains that have given numbers of lineages (*x*-axis). Solid lines, means. Shaded lines, SD.

In a recent study, we specified the lineage relationships between pairs of *Drosophila* peripheral neurons using a nucleus-targeting Bitbow1.0 (nBitbow1.0) that can generate 31 unique Bitbow codes (Veling et al., 2019). However, many more unique Bitbow codes are needed to unambiguously label the ∼200 neuronal lineages in the *Drosophila* central brain. We decided to label multiple subcellular compartments in the same fly to improve Bitbow’s coding capacity. We fused each FP to the N-terminal peptide from mouse Mannosidase II alpha 1 (mManII) to target the Golgi apparatus (Ye et al., 2007) as its morphology is distinct from the nucleus and the cell membrane. This resulted in a membrane/nucleus double-targeted mnBitbow1.0 fly and a membrane/nucleus/Golgi apparatus triple-targeted mngBitbow1.0 fly (Figure 2A), which can generate up to 1,023 and 32,767 (Figure 2B) unique Bitbow codes in the same brain, respectively.

To examine the lineage labeling efficacy, we crossed mnBitbow1.0 or mngBitbow1.0 flies to hsFlp;elav-Gal4 flies, and performed the larval heat-shock experiment (Figure 2C) to their offspring (Supplementary Figure 6, or Figure 2D, respectively). Similar to our previously reported nBitbow1.0 (Veling et al., 2019), mnBitbow1.0 or mngBitbow1.0 labeled many cell clusters, in which all the cells expressed the same combinatorial Bitbow code (Figures 2D–F). This indicated that the transient Flp activity turned on FP expression mostly in the neural stem cells and led to stable recombination outcome in the progenies. Many of these Bitbow codes contain FPs in more than one subcellular compartments, which indicates that the repeated incompatible FRT sites inserted in distant chromosome locations are exempt from inter-Bitbow cassette recombination. In addition, these subcellular compartments are spatially well separated, even when they are labeled by the same FPs in the same cell (Figure 2F).

To estimate the theoretical ability to unambiguously distinguish the 200 lineages in the same *Drosophila* central brain, we ran a “birthday problem” simulation to calculate the frequency of the same Bitbow code being seen in more than one lineage, i.e., the collision rate. The simulation shows a 84.5, 9.1, or 0.3% theoretical collision rate in a Bitbow fly that targets the five FPs to 1, 2, or 3 subcellular compartments, corresponding to 5-, 10-, or 15-bit Bitbow codes, respectively (Figure 2G dashed blue, green or red lines, respectively). In other words, under uniformly random recombination conditions, we can identify any neuron’s lineage composition in the mngBitbow1.0 fly central brain with 99.7% confidence. To estimate the collision rate in real experiments, we conducted the early heat-shock experiment as shown in Figure 2C with mnBitbow1.0 or mngBitbow1.0 flies. We plotted the percentages of cell clusters that are uniquely labeled, or 2 of them, or ≥3 of them are labeled by the same Bitbow code in each brain. We found that the experimental collision rates of mnBitbow and mngBitbow fly brains are 69.8% ± 5.7% (mean ± SD, 286 clusters from 4 brains) and 51.1% ± 8.5% (mean ± SD, 577 clusters from 6 brains), respectively (Figure 2H).

It seems desperate that the high collision rate would make even the mngBitbow1.0 fly useless for tracing neuronal lineages in the *Drosophila* central brain. However, we have shown that it is possible to develop a novel statistical method and apply it to the nBitbow1.0 flies to determine the lineage relationships between any two neighboring neurons in the *Drosophila* PNS (Veling et al., 2019). Given that the mngBitbow1.0 fly generates much more unique Bitbow codes, we sought a different strategy to simplify the analysis yet ensure proper statistical power to unambiguously trace any neuronal lineage composition in the *Drosophila* central brain. We plotted the percentages of Bitbow codes that are expressed in 1, or 2, or ≥3 clusters in each brain (Figure 2I). We found that the majority of labeling collisions were contributed by a small number of Bitbow codes that mostly have mNeonGreen being turned on, which also resulted in the mnBitbow and mngBitbow’s Shannon entropy being reduced to 5.9 and 8.5, respectively (Supplementary Figure 7). To estimate the effect of the FP turn-on bias to the apparent Bitbow code collision rates, we quantified the relative recombination frequencies of each FRT-FP module in mngBitbow1.0 (Supplementary Figure 8A), calculated the empirical frequencies of all 32,767 mngBitbow codes (Supplementary Figure 8B), and used the empirical frequencies to run the same “birthday problem” simulation as shown above (Figure 2G). We found that while mngBitbow1.0’s experimental collision rate was estimated as 40.3% for 200 lineages (Figure 2G, solid green line), a small number of codes appeared much more frequently and contributed to most of the collision events (Supplementary Figure 8C). When we excluded the most frequent 67 or 767 mngBitbow codes from the simulation, the collision rate decreased to 14.3 or 4.6%, respectively (Figure 2G, solid orange and purple lines). In other words, we have over 85.7 or 95.4% confidence to call any neurons belonging to the same lineage if only the pool of 32,700 or 32,000 lower frequent unique mngBitbow codes are used, respectively.

Encouraged by mngBitbow’s potential in determining lineage relationships with high confidence, we ran another simulation to estimate the number of animals needed to survey the lineage relationship of all neurons across the whole central brain, i.e., every one of the 200 lineages needs to be observed at least once (Figure 2J). We included the estimation for the popular method MARCM as a comparison (Lee and Luo, 1999). In the simulation, we assumed an average 48.08% lineage labeling rate for mngBitbow1.0 (577 clusters observed from six central brains containing an estimated total of 1,200 neuronal lineages) and a 1% lineage labeling rate for MARCM (to make sure no more than one lineage being labeled in each brain). This assumption underestimates the animal used in real MARCM experiments, that is because the same clonal patterns are normally required to be repeated more than once to confirm the labeling is indeed unique. Our simulation matches well with previous MARCM experiments (Yu et al., 2013; Lacin et al., 2014), in which hundreds to thousands of brains were needed in one experiment (Figure 2J, cyan line). Using mngBitbow1.0, only 28.3 ± 6.4 flies (mean ± SD) were needed to survey each of the 200 lineages at least once while achieving an overall >85% confidence in determining the lineage relationship between any neurons (Figure 2J, orange line).

### Bitbow2 Enables Broad Neuron Morphology Labeling With a Simple Transgenic Setup

While inducing Flp expression by heat-shock has the flexibility in controlling the timing of Bitbow1.0 recombination for lineage tracing, the relatively low Flp activity resulted in reduced color variation and labeling coverage, which constrains tracing morphology of postmitotic neurons. Increasing heat-shock duration to increase Flp activity was not ideal, because the animals were challenged by stronger stress, which resulted in a lower survival rate (data not shown). In addition, the requirement of heat-shocks limited the use of Bitbow in combination with other temperature-dependent interrogations (Kitamoto, 2001; Hamada et al., 2008). Finally, the hsFlp/enhancer-Gal4/Bitbow triple transgenes are more complicated to set up.

**FIGURE 3:**
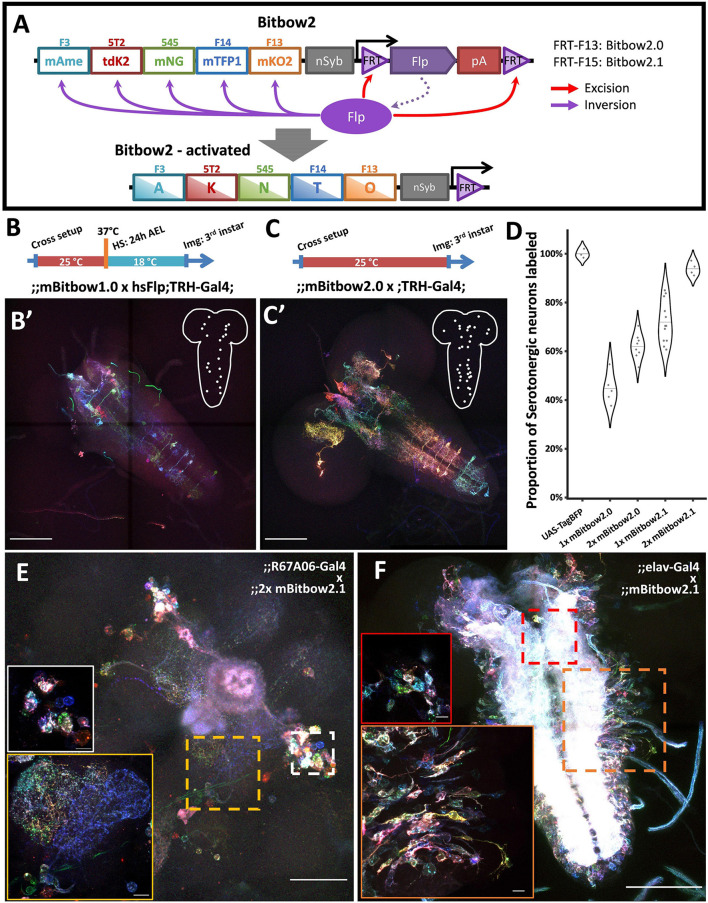
Bitbow2 enables broad neuron morphology labeling with a simple transgenic setup. **(A)** Schematic of Bitbow2 design. A self-regulating Flp (srFlp) module is added to ensure proper transient Flp activity without the need of an additional cross to the heat-shock Flp fly. Flp expression is driven by a neuron-specific n-Synaptobrevin (nSyb) promoter and terminated by self-excising between the flanking FRT sites, which have lower efficiency compared to those used in the Bitbow1 modules. This ensures proper Bitbow recombination before Flp self-excision to reach a stable genetic outcome. Compared to a **(B)** Bitbow1 labeling experiment, a **(C)** Bitbow2 labeling experiment requires only a direct cross to the TRH-Gal4 driver fly without the need of heat-shock. **(B’,C’)** indicate that mBitbow1.0 labeled fewer serotonergic neurons than Bitbow2.0 does. Inserted schematics indicate the somas of the labeled serotonergic neurons. **(D)** Quantification of the percentage of serotonergic neurons being labeled in different Bitbow2 flies, normalized to the labeling of a UAS-TagBFP fly. Each dot that overlays on the violin plots corresponds to the cell counting from one brain. **(E)** Adult neurons labeled in an offspring of the 2x mBitbow2.1 fly crossed to the R67A06-Gal4 fly. White and yellow insets show the somas of a group of neurons and their neurites projections, respectively. **(F)** Larva neurons labeled in an offspring of the 2x mBitbow2.1 fly crossed to the elav-Gal4 fly. Red and orange insets show one neuron cluster each in the central brain and in the VNC, labeled in distinct Bitbow colors, respectively. Dotted outline indicates the border of the neuron cluster. Scale bars: **(B’,C’,F)** 100 μm, **(E)** 50 μm, (**E,F**, inserts) 10 μm.

To overcome the above-mentioned limitations, we designed Bitbow2, in which a self-regulating Flp (srFlp) is added to Bitbow1 (Figure 3A). The srFlp consists of a flippase cDNA flanked by a pair of FRT sites positioned in the same direction. Driven by the promoter of *Drosophila* neuronal Synaptobrevin (nSyb; Riabinina et al., 2015), this design permits a strong burst of neuronal-specific expression of flippase which recombines the FP modules to generate Bitbow codes and eventually excises out the flippase cDNA to prevent chromosome breaks caused by excessive recombination. To ensure sufficient amount of flippase being produced before its coding sequence being removed, we made mBitbow2.0 and mBitbow2.1, which utilized the less efficient FRT-F13 and FRT-F15 sites to lower the chance of self-excision, respectively.

When using the TRH-Gal4 fly to label the ∼100 serotonergic neurons across the whole larva brain, we found that the labeling coverage of mBitbow1.0 is consistently outperformed by mBitbow2.0 (Figures 3B,B′,C,C′, respectively). Because FRT-F15 has an even lower recombination efficiency than FRT-F13, we hypothesized that mBitbow2.1 will have even better labeling coverage than mBitbow2.0 due to longer Flp activity before self-deactivation. In addition, we suspected that including two copies of mBitbow2 modules would have broader labeling coverage than the single copy counterparts due to more FP modules and stronger Flp activity. Indeed, we found that two copies of Bitbow2.1 generated the best labeling coverage, as high as 93.8% of all serotonergic neurons in a single fly (Figure 3D). When crossed to other subtype specific enhancer-Gal4 driver lines, Bitbow2 generated colorful labeling that recapitulated the classical UAS-myrGFP labeling (Figure 3E and Supplementary Figure 9). Finally, when crossing a Bitbow2.1 fly to an elav-Gal4 fly, its offspring labeled neighboring neurons in many distinct Bitbow colors, which indicates that Flp recombination is specific in postmitotic neurons (Figure 3F).

### Bitbow2 Enables Neural Anatomy and Network Analysis in the *Drosophila* Central Nervous System

As Bitbow2 provides rich color and broad coverage labeling, we expect it can be used to simultaneously resolve many neuron morphologies in the same brain. This not only increases the experimental throughput, but also eliminates the sampling errors and animal-to-animal variations in experiments that rely on aligning sparsely reconstructed neurons from multiple brains to a common reference (Peng et al., 2011). To be noted is that morphology labeling does not require using binary Bitbow codes to ensure the same lineage code being correctly registered for sibling neurons with differential FP expression levels. Instead, it is more important to have a consistent FP labeling across the whole cell membrane. In addition, including two copies of mBitbow cassettes in the same fly expands each FP’s possible range of expression level, which in turn enriches the color palette to allow better distinction between neighboring neurons.

**FIGURE 4:**
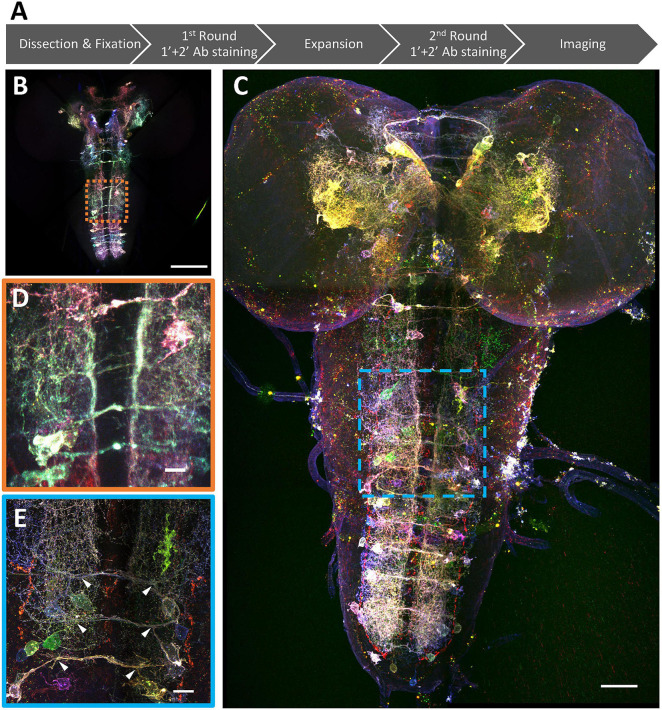
Super-resolution Bitbow imaging enabled by a modified protein retention-Expansion Microscopy (pro-ExM) protocol. **(A)** Experimental flow of the modified pro-ExM protocol. **(B)** Serotonergic neurons labeled by TRH-Gal4 driven 2x Bitbow2.1 without sample expansion and imaged by native fluorescence. **(C)** Serotonergic neurons labeled by TRH-Gal4 driven 2x Bitbow2.1 after ∼4x sample expansion and imaged by immuno-fluorescence. **(D,E)** Magnified boxed regions in panles **(B,C)**, respectively. Arrowheads indicate VNC serotonergic neurons within the same hemi-segment send out co-fasciculated neurites that form a single commissure projecting to the contralateral side. Scale bars, **(B,C)** 100 μm, **(D,E)** 10 μm.

**FIGURE 5:**
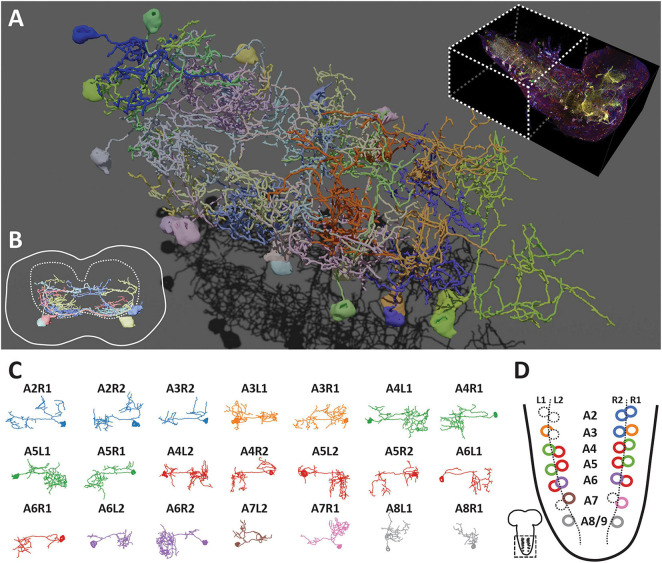
Bitbow2 tracing enables serotonergic neuron morphology and network analysis in larval VNC. **(A)** 3D rendering of traced VNC serotonergic neurons labeled by Bitbow2. Top right, dashed-line box indicates the VNC volume that has been traced. **(B)** Cross-section view of the traced serotonergic neurons located in the A5 segment (solid outline), which illustrates that the somas (solid oval shapes) are located at the very ventral part of the VNC while the neurites occupy mostly in the sensory zone (ventral part) of the neuropil (dashed outline). **(C)** Z-projections (dorsal view) of 21 traced serotonergic neurons categorized into 8 morphological subtypes that are indicated by distinct pseudo-colors. **(D)** Schematic of the abdominal VNC and serotonergic neurons. Circles indicate the soma locations of the traced serotonergic neurons and their colors correspond to the morphological subtype pseudo-colors in panel **(C)**. Dashed circles indicate the soma locations of the unlabeled serotonergic neurons.

We have previously shown that using protein-retention Expansion Microscopy (pro-ExM) can greatly enhance the imaging resolution to resolve closely the intermingled neurites in the dense neuropil of the mouse brain (Tillberg et al., 2016). Here, we applied a modified pro-ExM protocol to the Bitbow2 *Drosophila* brain (Figure 4). With ∼4x expansion, we could use nTracer (Roossien et al., 2019) to reconstruct all 21 Bitbow-labeled ventral nerve cord (VNC) serotonergic neurons [out of 26 estimated total (Chen and Condron, 2008; Huser et al., 2012)] from the A2 to A8/9 segments of a single 3rd instar larva brain (Figure 5A and Supplementary Movies 1, 2). We sampled the Bitbow colors along the somas and processes of these neurons and found that these 21 neurons were labeled by 16 well-separated colors in a UMAP projection (Supplementary Figure 10A). Although there were 3 instances where 2, or 2, or 4 neurons were labeled by very similar Bitbow colors (Supplementary Figure 10A, dash-line circles), their subtle color differences (Supplementary Figure 10B) and well-separated physical locations (Supplementary Figure 10C) allowed us to easily distinguish them from their neighbors. In addition, we found the soma and neurites of these serotonergic neurons were labeled in consistent Bitbow colors, which permitted us to reconstruct their morphology with little ambiguity (Supplementary Figures 10D,E). We found that all VNC serotonergic neurons project quite locally, mostly within the same segment (Figure 5A). Their somas are located at a very ventral part of the VNC and their projections are mostly restricted to the sensory zone (ventral half) of the VNC (Figure 5B; Sykes and Condron, 2005; Chen and Condron, 2008).

As the majority of serotonergic neurons in the A2 to A8/9 segments of this VNC were labeled and reconstructed, we paid extra attention to discover potential anatomical roles that respect the repeated hemi-segment patterns of the VNC (Supplementary Figure 11). We noticed that all VNC serotonergic neurons within the same hemi-segment send out co-fasciculated neurites that form a single commissure projecting to the contralateral side (Figure 4E, arrowheads). While serotonergic neurons in the same hemi-segment have quite distinct morphologies and projection patterns, they have similar counterparts in the contralateral hemi-segment, therefore, forming a bi-lateral symmetric network (Figures 5C,D). These morphologically similar neurons can be classified as at least eight distinct subtypes based on the quantification of morphological features, including projection density in the contralateral and the ipsilateral side, major branching patterns and anterior vs. posterior projection distribution (Figure 5D, detailed in the section “Materials and Methods”).

## Discussion

We reported Bitbow, a set of novel transgenic tools capable of generating a large number of unique imaging barcodes in a single animal (Table 1). Bitbow utilizes a novel design, in which independent Flp/FRT recombination events lead to binary choices of expressing orthogonal spectral labels. This mechanism exponentially expands the color-coding capacity to 2^N^−1 when using N “bits” of spectrally distinguishable tags. Targeting the same 5-FPs to 3 imaging differentiable subcellular compartments, we created mngBitbow1.0 transgenic flies, which can generate up to 32,767 unique Bitbow codes in a single brain. This is a significant advantage for imaging-based lineage tracing studies because it greatly increases the possibility of labeling neurons with unique lineage codes. Interestingly, we found that heat-shock induced recombination events are constrained in neural stem cells of the larval Bitbow flies. Such serendipity permits directly using Bitbow codes to determine lineage relationships between neural progenies. Providing statistical quantification and modeling, we established that it is feasible to map the lineage relationships between any subtype-specific neurons, driven by any enhancer-Gal4, using as few as ∼10 brains.

**TABLE 1:** Transgenic *Drosophila* Bitbow summary.

**Bitbow version**	**Subcellular compartment**	**Insertion site (Chr.)**	**srFlp**	**Labeling density**
mBitbow 1.0	Cell membrane	attP40(2L) or attP2(3L)	No	Variable (with hsFlp)
nBitbow 1.0	Nucleus	attP40(2L) or VK27(3R)	No	Variable (with hsFlp)
gBitbow 1.0	Golgi	attP40(2L) or attP2(3L)	No	Variable (with hsFlp)
mBitbow 2.0	Cell membrane	attP2(3L) or VK27(3R)	Yes	Medium (40–60%)
mBitbow 2.1	Cell membrane	attP2(3L) or VK27(3R)	Yes	High (70–95%)

*Chr., Chromosome location, srFlp, self-regulating Flp.*

In practice, we found that certain FP expressed with much higher frequencies than other ones. We suspect that this is due to more frequently spinning of their flanking FRT sites under suboptimal recombination conditions, such as heat-shock induced transient Flp activity. Bitbow codes containing these FPs would have higher collision rates when used in lineage mapping studies. We mitigate such disadvantages by excluding cells labeled by the high-frequency Bitbow codes from analysis. In the future, this problem can be avoided by screening more incompatible FRT sites and using only those with similar recombination efficiencies. Nonetheless, there are other disadvantages associated with heat-shock induced Flp recombination, especially for neuronal morphology labeling and reconstruction. We found a low percentage of cells were being labeled by heat-shock induced mBitbow expression. In addition, the generated Bitbow colors were relatively simple in a way that neurons labeled by more than two FPs were relatively rare.

To solve the above-mentioned problems for morphology labeling, we generated Bitbow2 transgenic flies, in which a novel srFlp module was integrated to effectively recombine mBitbow1 without the need of heat-shock. The elimination of the *hs*-Flp allele yielded two additional advantages: (1) Needing only a simple cross to the broadly used Gal4 libraries, Bitbow2 can be used as a drop-in replacement to any UAS-FP reporters. (2) Abolishing the need for heat-shock, Bitbow2 is compatible with temperature-sensitive assays, such as heat-induced neuronal manipulations with shibire^ts^ (Kitamoto, 2001), dTrpA1 (Hamada et al., 2008), etc. Finally, we generated different versions of Bitbow2 flies, each of which labeled a different percentage of total neurons to suit the need of tuning the labeling density for different Gal4 driver lines. It would be also worth mentioning that among the Bitbow2 flies, we found that 2-copy transgenic flies (2xmBitbow2.0 or 2xmBitbow2.1) had better labeling coverage than their 1-copy counterparts, which was greatly beneficial for more complete morphological reconstructions. In this case the Bitbow color outputs are no longer binary, instead, it expands each FP’s possible range of expression level, which in turn enriches the color palette to allow better distinction between neighboring neurons.

Combining sample expansion (ExM) and saturated neuron tracing (nTracer), Bitbow2 flies are suitable for high-throughput morphology studies from light microscopy images. We found that Bitbow labeling is statistically consistent throughout the neuron soma and neurites. This builds the confidence of using fluorescence intensity difference in each spectral channel to differentiate neighboring neurons when using nTracer to reconstruct their morphology. We estimate that thousands (∼5^5^) of Bitbow “colors” can be easily distinguished in a well-taken 16-bit image dataset. Densely packed neurons, such as the VNC serotonergic neurons are now readily traceable to not only classify the morphological heterogeneity, but also reveal the neural network patterns among a genetically defined population. We envision that, in the future, combining with other high-throughput modalities, such as light-sheet microscopy and automated neuronal tracing, will make larger scale, multi-brain morphological studies feasible in most laboratories.

## Materials and Methods

### Key Resources

**Table:** 

**Reagent or Resource**	**Source**	**Identifier**
**Antibodies**		
Rat Anti-mTFP	Cai Lab	
Chicken Anti-GFP	Cai Lab	
Rabbit Anti-mNeonGreen	Cai Lab	
Mouse Anti-mKusabira-Orange2	Cai Lab	
Guinea Pig Anti-mKate2	Cai Lab	
Alexa Fluor 594 Donkey Anti-Rat	Life Technologies	A21209
Alexa Fluor 488 Donkey Anti-Chicken	Jackson ImmunoResearch	703-545-155
Atto 490LS Goat Anti-Rabbit	Hypermol	2309
CF 555 Donkey Anti-Mouse	Sigma	SAB4600060
CF 633 Donkey Anti-Guinea Pig	Sigma	SAB4600129
Alexa Fluor 647 Donkey Anti-Guinea Pig	Jackson ImmunoResearch	706-605-148
**Bacterial and Virus Strains**		
Mach1-T1 Chemically Competent *E. coli*	Thermo Scientific	C862003
Stbl3 Chemically Competent *E. coli*	Thermo Scientific	C737303
**Chemicals, Peptides, and Recombinant Proteins**		
Acrylic acid N-hydroxysuccinimide ester	Sigma	A8060
Sodium Acrylate	Sigma	408220
Acrylamide	Sigma	A9099
N,N’-methylenebisacrylamide	Sigma	M7279
Tetramethylethylenediamine (TEMED)	Sigma	T7024
Ammonium Persulfate	Sigma	A3678
4-hydroxy-2,2,6, 6-tetramethylpiperidin-1-oxyl (4HT)	Sigma	176141
Startingblock (PBS) blocking buffer	Thermo Scientific	37578
Proteinase K	NEB	P8107
**Experimental Models: Cell Lines**		
*D. melanogaster*: S2	Bing Ye Lab	
*M. musculus*: Neuro-2a	ATCC	CCL-131
**Experimental Models: Organisms/Strains**		
*D. melanogaster*: w1118;;	BDSC	RRID: BDSC_5905
*D. melanogaster*: hsFlp112;;	BDSC	
*D. melanogaster*: hsFlp112;Sp/CyO;TM2,Ubx/TM6B,Tb	Bing Ye Lab	
*D. melanogaster*: hsFlp112;;elav-Gal4	This study	
*D. melanogaster*: hsFlp112;TRH-Gal4;	This study	
*D. melanogaster*: yw;;mBitbow1.0	This study	
*D. melanogaster*: yw;;nBitbow1.0	This study	
*D. melanogaster*: yw;gBitbow1.0;	This study	
*D. melanogaster*: yw;gBitbow1.0/CyO;mBitbow1.0, nBitbow1.0	This study	
*D. melanogaster*: yw;;mBitbow2.0	This study	
*D. melanogaster*: yw;;mBitbow2.1	This study	
*D. melanogaster*: yw;;2x[mBitbow2.0]	This study	
*D. melanogaster*: yw;;2x[mBitbow2.1]	This study	
*D. melanogaster*: w;elav-Gal4/CyO;	BDSC	8765
*D. melanogaster*: w;TRH-Gal4;	BDSC	38388
*D. melanogaster*: w;;R53C10-Gal4	BDSC	38873
*D. melanogaster*: w;;R67A06-Gal4	BDSC	39397
**Recombinant DNA**		
pmAmetrine-N1	Addgene	54505
pmTFP1-N1	Addgene	54521
pmNeonGreen-N1	Allele Biotechnology	
pmKusabira-Orange2-N1	Sakaue-Sawano et al., 2008	
ptdKatushka2-N	Shcherbo et al., 2009	
pmCitrine-N1	Addgene	54594
pmCherry-N1	Shaner et al., 2004	
pJFRC-MUH	Addgene	26213
pattB-synaptobrevin-GAL4-hsp70	Addgene	46107
pJFRC81-10XUAS-IVS-Syn21-GFP-p10	Addgene	36432
pMT-Gal4	DGRC	1042
pUAST-Flp	DGRC	1020
pCAG-FlpINT	Dawen Cai Lab	
pDC-MUH	This study	
pDC-MUH-p10pA	This study	
pCMV-2xmAmetrine-N	This study	
pDC-UAS-mBitbow1.0	This study	
pDC-UAS-nBitbow1.0	This study	
pDC-UAS-gBitbow1.0	This study	
pDC-UAS-mBitbow2.0	This study	
pDC-UAS-mBitbow2.1	This study	
pAc5-Flp-p10pA	This study	
pCMV-3FRT-mCherry-F14-mTFP	This study	
pCMV-3FRT-mCherry-F15-mCit	This study	
pCMV-3FRT-F14-mTFP-F15-mCit	This study	
pCMV-3FRT-F14-F15-mCit-F13-mCherry	This study	
pCAG-Flpbow3	Cai et al., 2013	
**Software and Algorithms**		
Fiji	Schindelin et al., 2012	https://fiji.sc/
nTracer (Fiji plugin)	Roossien et al., 2019	https://www.cai-lab.org/ntracer-tutorial
Spectral Unmixing (Fiji plugin)	Joachim Walter	https://imagej.nih.gov/ij/plugins/spectral-unmixing.html
Lasergene	DNASTAR	https://www.dnastar.com/
Graphpad Prism	Graphpad Software	https://www.graphpad.com/
Vaa3D	Peng et al., 2010	http://home.penglab.com/proj/vaa3d/home/
Blender	Blender Foundation	https://www.blender.org/
NumPy	NumPy.org	https://numpy.org/
umap-learn	McInnes et al., 2018	https://github.com/lmcinnes/umap

### *Drosophila* Husbandry

Flies were reared at 25°C on standard CT food with a 12 h/12 h light/dark cycle. For heat-shock induced Bitbow labeling experiments, hsFlp;elav-Gal4 or hsFlp;TRH-Gal4; females were crossed to Bitbow1.0 males, and timed-egg-lay was conducted to collect embryos for the desired time window in vials; afterward, the vials were placed in a 37°C metal-bead bath for 30 min to induce the heat-shock, and kept at 18°C to incubate until ready for dissection.

### Molecular Cloning and Fly Transgenics

To test out new incompatible FRT sites, a series of FRT-FP plasmids were constructed (Supplementary Figure 1) using the mammalian expression backbone pCMV-N1 (Clontech). FRT-F13, FRT-F14, and FRT-F15 sequences were obtained from a previous study (Turan et al., 2010), and introduced to the following plasmids through PCRs. To test the incompatibility of FRT-F14 and FRT-F15 to three known FRTs (FRT-F3, FRT-5T2, and FRT-545), pCMV-3FRT-mCherry-F14-mTFP and pCMV-3FRT-mCherry-F15-mCit were built through sequential assembly of the three FRTs from pCAG-Flybow (Cai lab), mCherry-SV40pA from pmCherry-N1 (Addgene), and F14-mTFP-SV40pA or F15-mCitrine-SV40pA from pmTFP-N1 or pmCit-N1 (Addgene), respectively, using PCR, restriction digestion, and ligation. After the incompatibility test was done, mCherry-pA was removed by digestion with two flanking blunt-end sites (*Pml*I, *Eco*RV), and re-ligation to produce pCMV-3FRT-F14-mTFP and pCMV-3FRT-F15-mCit, as the control plasmids. Similar cloning approaches were applied in the next steps. To test incompatibility of FRT-F15 to the other four FRT sites, F15-mCit-pA was moved into pCMV-3FRT-F14-mTFP to produce pCMV-3FRT-F14-mTFP-F15-mCit, from which mTFP-SV40pA was removed to produce the control plasmid pCMV-3FRT-F14-F15-mCit. Finally, to test incompatibility of FRT-F13 to the other five FRT sites, F13-mCherry-pA was moved into pCMV-3FRT-F14-F15-mCit to produce pCMV-3FRT-F14-F15-mCit-F13-mCherry, from which mCitrine-SV40pA was removed to produce the control plasmid pCMV-3FRT-F14-F15-F13-mCherry.

For Bitbow1 plasmids, cDNAs encoding the following FPs were used: mAmetrine, mTFP, mNeonGreen, mKusabira-Orange2, and tdKatushka2 (Ai et al., 2006, 2008; Sakaue-Sawano et al., 2008; Shcherbo et al., 2009; Shaner et al., 2013). *Drosophila* myristoylation signal peptide of dSrc64B (1-10aa, dMyr), Human histone 2B protein (full length, hH2B) or Mouse Mannosidase II alpha 1 (1-112aa, mManII) was fused in-frame to the N-terminus of individual FPs (dMyr-FP, hH2B-FP, mMannII-FP), to achieve targeted labeling at the cell membrane, nucleus or Golgi apparatus (Kanda et al., 1998; Struhl and Adachi, 1998; Ye et al., 2007). Individual incompatible FRT sequence pairs (FRT-F13, FRT-F14, FRT-545, FRT-F3, or FRT-5T2) (McLeod et al., 1986; Volkert and Broach, 1986; Schlake and Bode, 1994; Turan et al., 2010; Cai et al., 2013) were then placed in the opposing direction to flank dMyr-FP/hH2B-FP/mMannII-FP sequence. FP-FRT pairings were: mAmetrine – F3, tdKatushka2 – 5T2, mNeonGreen – 545, mTFP1 – F14, and mKusabira-Orange2 – F13 (Figure 1A). An upstream activation sequence (UAS) and a p10 polyadenylation sequence (p10pA) (Pfeiffer et al., 2012) were placed upstream and downstream of each FRT flanked FP cassette, respectively, and separately cloned into pDC-MUH, which was based on the pJFRC-MUH backbone vector (Pfeiffer et al., 2010) with a few digestion site modifications, by standard cloning methods. The Bitbow1.0 plasmids were finally assembled together from the five individual modules through Gibson assembly (Gibson et al., 2009).

For Bitbow2 plasmids, the nSyb-promoter-driving self-regulating flippase module was constructed by flanking FlpINT [flippase with an inserted *c. elegans* intron (Davis et al., 2008), Cai lab] cDNA with a FRT-F13 pair or a FRT-F15 pair which were oriented in the same direction, and then placed downstream of a *Drosophila* n-Synaptobrevin promoter (Riabinina et al., 2015). The module was then inserted into the mBitbow1.0 plasmid, at a location far away from all 5 FP modules, through Gibson Assembly to generate mBitbow2.0 or mBitbow2.1.

The final Bitbow plasmids were integrated into *Drosophila melanogaster* genome docking sites attP40, attP2 or VK00027 (Table 1) through ΦC31-integrase-mediated transgenesis (Groth, 2004; Bateman et al., 2006; Venken et al., 2006; Bischof et al., 2007; Markstein et al., 2008). Embryo injections and transgenic selections were done by BestGene Inc., (Chino Hills, CA, United States).

### Dissection and Mounting

Adult or 3rd instar *Drosophila* brains were dissected in PBS at room temperature (abbr. RT) within 30 min before proceeding to fixation. Dissected brains were fixed in 4% PFA (Sigma #P6148, diluted in PBS) at RT with gentle nutation for 20 min, followed by three quick PBST (PBS + 1% Triton X-100) washes, then PBS washes for 15 min × 3. Brains then either proceeded to direct mounting (for native fluorescence imaging) or immuno-stainings. Vectashield (Vector Laboratories, H-1000) was used as the mounting medium.

### Immunohistochemistry

Fixed brain samples were treated with StartingBlock (Thermo, 37578) for 1 h at RT with gentle nutation. After blocking, the brains were incubated with primary antibodies diluted in StartingBlock for 2 overnights at 4°C. Three quick PBST washes and PBS washes for 15 min × 3 were done, before the brains were incubated with secondary antibodies diluted in StartingBlock for 2 overnights at 4°C. Finally three quick PBST washes and PBS washes for 15 min × 3 were done and the brains were ready for imaging. For detailed antibody combinations and dilutions see section “Key Resources.”

### Expansion Microscopy

Expansion microscopy brain samples were generated following the pro-ExM protocol (Tillberg et al., 2016) with modifications. Antibody-stained Bitbow samples were treated in Acrylic acid N-hydroxysuccinimide ester (AaX, Sigma, A8060) at RT for 1 overnight, followed by PBS washes for 15 min × 3. Samples were then incubated in the ExM monomer solution (“Stock-X,” containing Acrylate, Acrylamide, and Bis-acrylamide) at 4°C for 1 overnight. Samples were transferred to fresh ExM monomer solution with gel initiators (APS, TEMED, and 4-HT) at 4°C for 15 min, and then quickly mounted on a sample chamber made with 200 μm adaptors (Sun lab) on a glass slide, sealed with a 22 × 30 coverslip on top (Fisher, 12-544). The slide was then transferred to a humidity box and incubated at 37°C for about 2 h until the gel fully polymerized. The gel was trimmed carefully with a razor to allow as little of excessive space around the brains as possible. Trimmed gel pieces were transferred to an EP tube and digested with Proteinase K (NEB, P8107) at 37°C for 1 h. Three quick PBST washes and PBS washes for 15 min × 3 were done before the brains were put into another round of antibody staining, following the same IHC protocol mentioned above. After the second-round staining, the gels were slowly expanded to the final size by changing the submerging solution from PBS to pure diH2O, and ready for imaging.

### Confocal Microscopy and Linear Unmixing

Confocal images were acquired with Zeiss LSM780 with a 20 × 1.0 NA water immersion objective (421452-9800-000) or a 40 × 1.3 NA oil immersion objective (421762-9900-000). The 32-channel GaAsP array detector was used to allow multi-track detection of five fluorophores with proper channel collection setups (Supplementary Figure 2).

Spectral Unmixing plug-in (by Joachim Walter) in Fiji was used to perform linear unmixing on Bitbow images. Reference unmixing matrix was measured by imaging cultured mouse N2A cells expressing mAmetrine, mTFP, mNeonGreen, mKO2, or tdKatushka2 separately, with the exact same multi-track setups intended for Bitbow brains (Supplementary Figure 2). Customized ImageJ scripts were used to automate the unmixing process as well as creating composite image stacks from unmixed channels. ImageJ scripts and corresponding unmixing matrices can be acquired from our Github repository^1^.

### Image Stitching and Neuron Tracing

When the region of interest was larger than the objective field of view, multiple confocal tiles were taken and stitched offline. 5% overlapping seams were set between adjacent tiles to allow reliable stitching and maximize the area of coverage. Alignmaster 1.0.6 (part of the nTracer tool set) was used to perform stitching between tiles sequentially.

All neuronal tracings were done using nTracer 1.3.5. Sampling tolerance for color and intensity were set at 0.3 to allow accurate and efficient tracings. Tracing results were exported in SWC format for downstream 3D-rendering and Bitbow color analysis.

3D visualizations of neuron tracings were performed using custom scripts in the open-source modeling software Blender v2.81 (Blender Foundation). Models containing fluorescence data were produced with a modification of the method described in Haschka et al. (2015).

### Quantification and Statistical Analysis

#### Bitbow Code Quantifications

Bitbow-labeled neural clusters in the 3rd instar larval brains were used to quantify the labeling performance of 1-localization, 2-localization, and 3-localization Bitbows. Clusters in the central brain, gnathal segments, and thoracic segments were marked with the Fiji ROI tool, and the on/off status of each color channel in every cluster was manually recorded as 1/0 for each “bit” (examples in Figures 1E, 2F). The frequency of occurrence of each Bitbow module was calculated in each brain, and summarized across multiple brains with the mean and standard deviation of the frequency reported (Figure 1H and Supplementary Figure 8A). 15-module frequencies of mngBitbow were used to generate empirical probability distribution of all 32,767 mngBitbow codes, which was further used in simulations described in Figures 2G,J (details below).

#### Theoretical and Experimental Bitbow Barcode Collision Rates

Calculation of the theoretical collision rates (Figure 2G) was done in a similar way to solve the ‘‘birthday problem.’’^2^ First the expected number of collisions c was calculated using a closed-form formula, with n as the number of coded lineages, and b as the number of Bitbow codes:

c = n − b ^∗^ (1 − ((b−1)/b)^∗∗^n)

Then, the collision rate was produced by dividing c by n.

Simulations of the experimental (empirical) collision rates (Figure 2G) were performed in a similar fashion. In brief, random lists of barcodes were drawn from the empirical distributions, followed by the counting of repeated barcodes to produce an overlap rate. This process was repeated 1,00,000 times for systems under 100 lineages, 1,000 times for systems between 100 and 1,000 lineages, and 10 times between 1,000 and 1,000,000 lineages, due to computational complexity.

#### Estimation of the Number of Bitbow Fly Brains Needed to Label All Central Brain Lineages at Least Once

Computer simulations were used to estimate the number of animals required to achieve saturated coverage for a range of hypothetical N-neuron systems (Figure 2J). For each condition, 500 trials of Algorithm 1 were averaged using custom code implemented in Python v3.7.4 and NumPy v1.17.2. Here, *activation_rate* was assigned to estimates of 0.5% (MARCM) and 48.08% (Bitbow) to model differences in labeling densities. The whitelist array *valid_barcodes* was assigned as the k lowest-probability barcodes (32,767, 32,700, or 32,000) for the Bitbow trials or all possible barcodes for MARCM.

**ALGORITHM 1:**
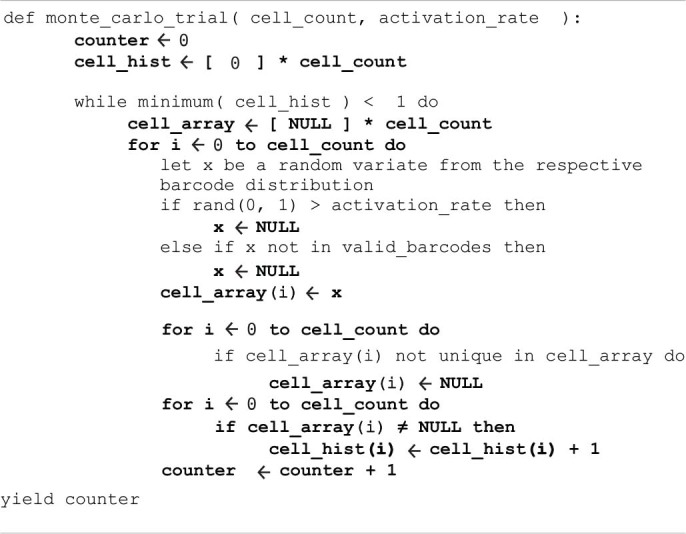
Monte Carlo simulation to estimate the number of animals needed for sampling a given number of cells at least once.

#### Analysis of mBitbow Color Separation Between Different Neurons and Labeling Consistency Within the Same Neurons

Pixel intensity values from 5 channels along the tracing of all 21 somas and part of 4 neurites (A5L1, A5L2, A5R1, and A5R2) were used to generate analysis on differentiation power as well as the stability of Bibtow labeling. Raw intensities were processed through a 3 × 3 × 3 median kernel and a 10-pixel rolling window average to reduce noise, then the pixel intensity in each channel was normalized to the sum of five channels of that pixel, in order to bring brighter and dimmer pixels to the same scale for accurate color analysis.

To visualize the color separation between 21 neuron somas, UMAP was used to project the intensity values of all five channel dimensions onto a 2D display (Supplementary Figure 10A). To visualize the color differences between the 4 physically closely located neurons, a separate UMAP was generated using the same parameters (Supplementary Figures 10B,C). Data were processed with Python 3.7.4 and umap-learn 0.3.10.

To visualize the consistency of Bitbow labeling, soma pixel intensities and neurite pixel intensities from the same neurons were summarized in “split-violin” plots, where in each plot the left half represents soma pixels and the right half neurite pixels (Supplementary Figure 10E).

## Data Availability Statement

The datasets presented in this study can be found in online repositories. The names of the repository/repositories and accession number(s) can be found in the article/Supplementary Material.

## Author Contributions

YL and DC conceived the project and designed the experiments. YL, LW, and DC wrote the manuscript with input from all authors. YL designed the cloning strategies to construct Bitbow plasmids and wrote the codes for Bitbow color analysis. YL, YZ, MG, TC, and DR generated the Bitbow transgenic flies. YL, YZ, and EE processed the brain samples and performed the microscopy. YL and MC quantified the Bitbow1 lineage codes. YL and EE quantified the Bitbow2 labeling coverage. YL and HC traced the VNC serotonergic neurons. LW, YL, NM, and DC established the statistical models. LW wrote the codes for simulations and scripts for 3D renderings of traced neurons. DC initiated and supervised the project. All authors contributed to the article and approved the submitted version.

## Conflict of Interest

The authors declare that the research was conducted in the absence of any commercial or financial relationships that could be construed as a potential conflict of interest.

## Publisher’s Note

All claims expressed in this article are solely those of the authors and do not necessarily represent those of their affiliated organizations, or those of the publisher, the editors and the reviewers. Any product that may be evaluated in this article, or claim that may be made by its manufacturer, is not guaranteed or endorsed by the publisher.

## Footnotes

1
https://github.com/Cai-Lab-at-University-of-Michigan

2
https://en.wikipedia.org/wiki/Birthday_problem#Number_of_people_who_repeat_a_birthday
