# Supplementary material for: Bitbow Enables Highly Efficient Neuronal Lineage Tracing and Morphology Reconstruction in Single Drosophila Brains
Source: Front Neural Circuits. 2021 Oct 20;15:732183. doi: 10.3389/fncir.2021.732183 (PMC8564373; doi:10.3389/fncir.2021.732183)
Supplement: Supplementary file 3 [file Data_Sheet_1.PDF]

Supplementary Material

1 Supplementary Figures

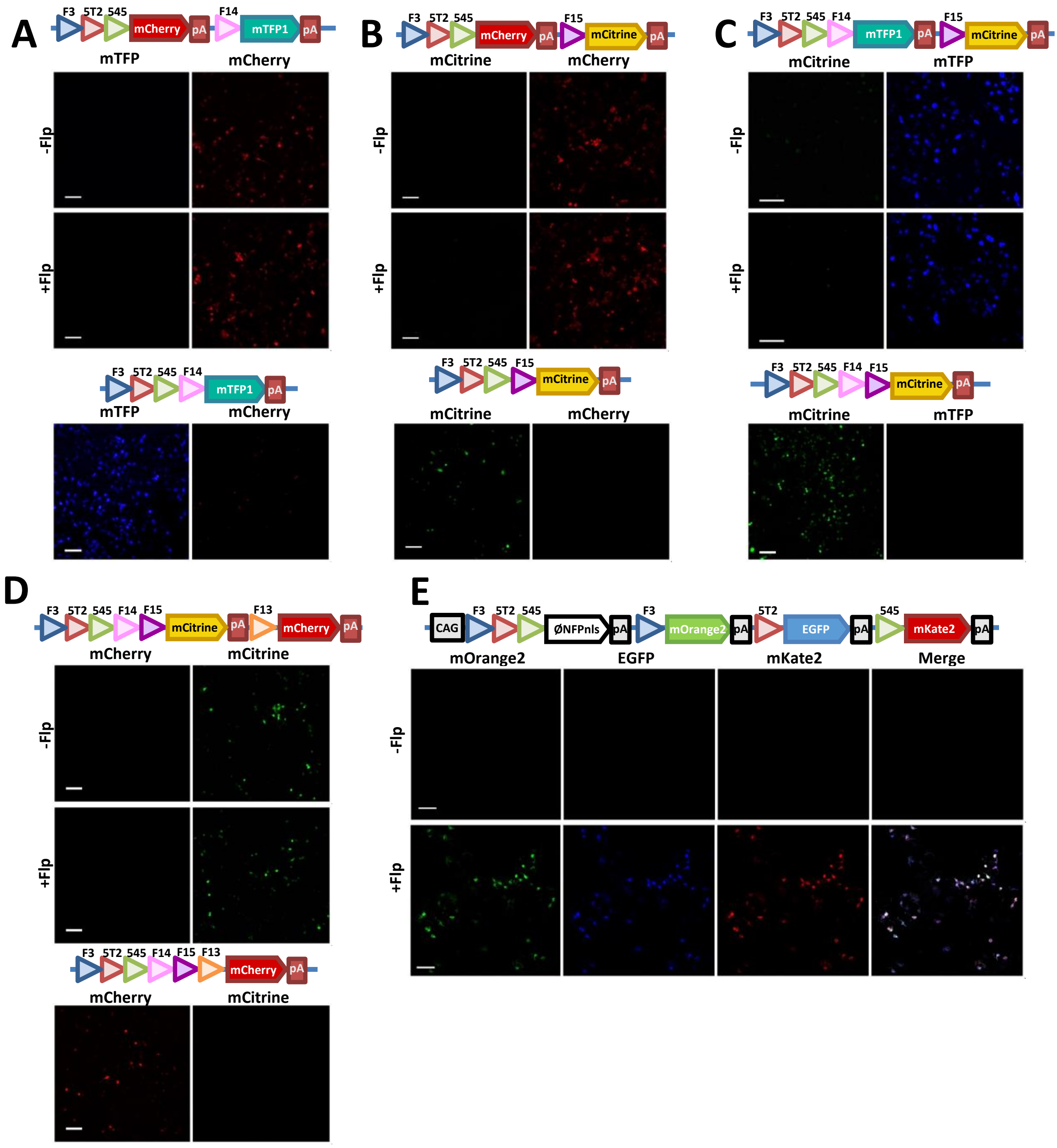

Figure S1. Validation of additional orthogonal FRT sites to be used in the Bitbow constructs. Series of constructs were generated and co-transfected with pCAG-Flp into mouse N2A cells to determine the orthogonality of FRT variants in addition to the previously reported FRT-F3 (blue triangles), FRT-5T2 (green triangles) and FRT-545 (red triangles) sites (3FRT). **(A, B)** show the

results of validation of FRT-F14 (pink triangles) and FRT-F15 (magenta triangles), respectively. In either experiment, mCherry expression was driven by the CMV promoter and the mRNA was terminated by a polyadenylation (pA) sequence positioned right after the mCherry. If the FRT-F14 or FRT-F15 site can be recombined with any of the 3FRT sites, the removal of mCherry-pA sequence would result in the expression of mTFP or mCitrine, respectively. We found that regardless of whether Flp is co-expressed or not, only mCherry expression was observed, which indicated the FRT-F14 or FRT-F15 site is incompatible to the 3FRT sites (top panel of A or B, respectively). Next, we used restriction enzymes to remove the mCherry-pA sequence and found that mTFP or mCitrine can indeed be properly expressed if this was caused by Flp/FRT recombination (bottom panel of A or B, respectively). **(C)** To validate whether FRT-F14 and FRT-F15 are incompatible to each other, we took the pCMV-3FRT-F14-mTFP-pA construct from (A) and concatenated F15-mCitrine-pA to its 3’-end. We found that regardless of whether Flp is co-expressed or not, only mTFP expression was observed, which indicated that FRT-F14 is incompatible to FRT-F15. (C, top panel). And similarly, we generated a 5FRT-mCitrine-pA plasmid and confirmed the proper expression of mCitrine if mTFP-pA sequence was removed (C, bottom panel). **(D)** To validate whether FRT-F13 is incompatible to the other 5 FRT sites, we took the 5FRT-mCitrine-pA and concatenated F13-mCherry-pA to its 3’-end. We found that regardless of whether Flp is co-expressed or not, only mCitrine expression was observed, which indicated that FRT-F13 is a 6th incompatible FRT site. (D, top panel). And again, we generated a 6FRT-mCherry-pA plasmid and confirmed the proper expression of mCherry if mCitrine-pA sequence was removed (D, bottom panel). **(E)** The activity of Flp in experiments (**A-D**) was demonstrated by including a control, in which the pCAG-Flpbow3 plasmid (Cai *et al.* 2013) was co-transfected with or without a pCAG-Flp plasmid.

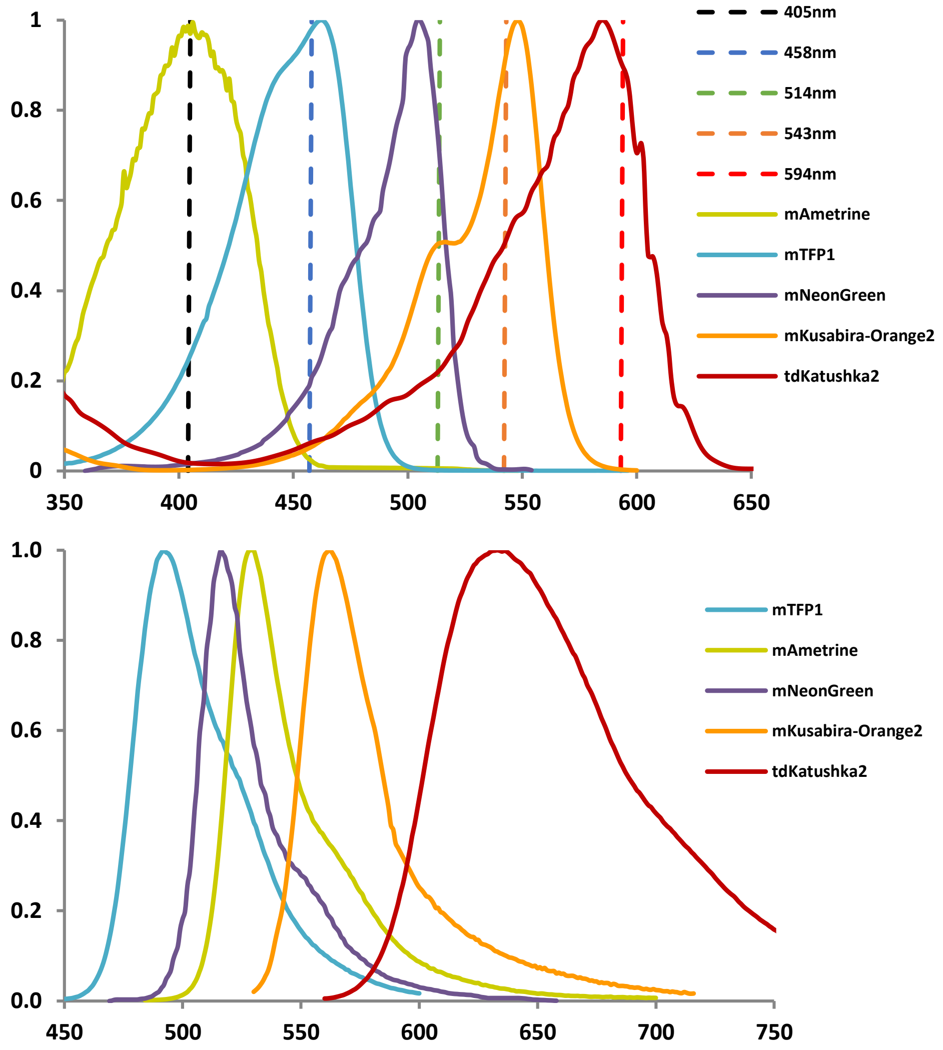

Track	MBS	Laser	Channels (Fluorophores)
I	-405/458/514/594	405nm	456-501(mTFP*), 501-545(mAme)
II	-405/458/514/594	458nm	456-501(mTFP), 501-545(mNG*)
III	-405/458/514/594	514nm, 594nm	501-545(mNG), 545-572(mKuO2), 625-696(tdKatu2)

Figure S2. Spectral property and confocal imaging settings for the five Bitbow fluorescent proteins (FPs). Top panel, normalized absorbance (excitation) efficiency spectra of the five FPs. Dashed vertical lines, wavelength of excitation lasers used to image the five FPs. Middle panel, normalized emission efficiency spectra of the five FPs. Bottom panel, confocal microscope setup to sequentially image these FPs in line-switching mode. MBS, main beamsplitter. Asterisks, extra recording channels used for spectral linear unmixing (detailed in **Methods**).

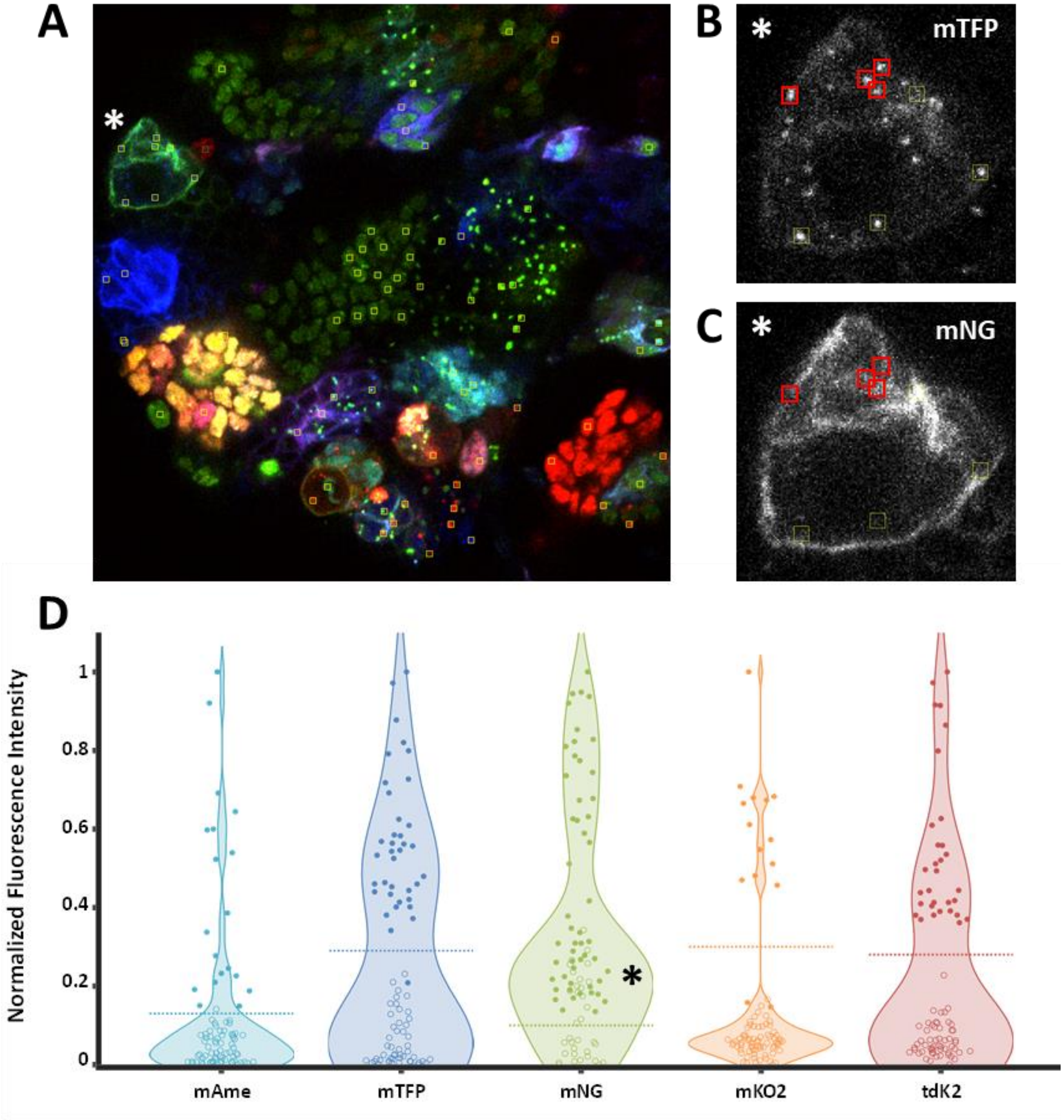

Figure S3. Bitbow fluorescence yields reliable binary output. (A) image plane from a mngBitbow brain that was shown in **Figure 2E'**. Yellow squares indicate the regions of interest (ROIs) being sampled to measure the fluorescence intensities in each spectral channel for binary Bitbow code calling. **(B, C)** mTFP and mNG channel images, respectively, of the asterisk-marked cell in (A). Squares marked out corresponding ROIs for binary Bitbow code calling. **(D)** Violin plots of normalized fluorescence intensity readout from each sampling ROI, in all five channels. Dashed lines indicate the normalized intensity cut-offs for calling "ON" or "OFF" in each Bitbow channel (N=86). Solid circles, data points corresponding to "ON" in Bitbow code calling. Open circles, data points corresponding to "OFF". Asterisk indicates multiple "OFF" calling ROIs in the mNG channel that have greater intensity than the cut-off. Representatives of these ROIs are shown as the red boxes in (B, C). As we can see that while the Golgi apparatus is clearly marked in the mTFP channel and non-exist in the mNG channel, the same ROI positions in the mNG channel convolute adjacent membrane labeling, which results in elevated intensity.

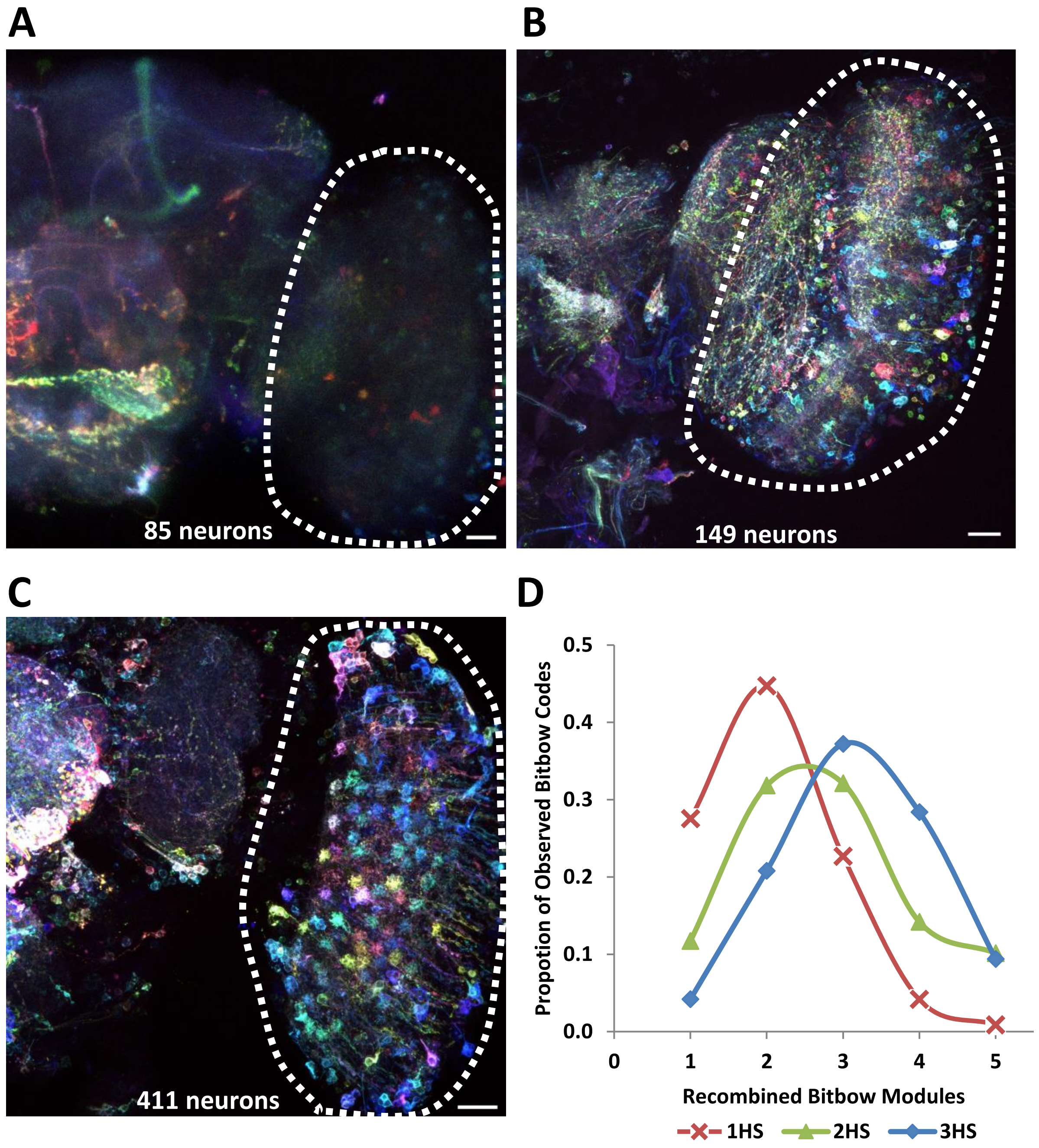

Figure S4. Multiple heat-shock induced flippase activity labels more neurons in mBitbow1.0 flies. Adult fly brains from a hsFlp;;elav-Gal4 to mBitbow1.0 cross were experimented with **(A)** one, **(B)** two or **(C)** three 30-minute heat-shocks, similar to the protocol shown in **Figure 1C** Neuron numbers were counted in the dash line defined regions in the full image stack. Scale bars are 20μm. **(D)** Distribution of Bitbow codes observed in 1-HS, 2-HS and 3-HS samples, regarding how many modules were seen to be "ON". For example, both Bitbow codes "10101" and "11010" have 3 "ON" modules and 2 "OFF" modules.

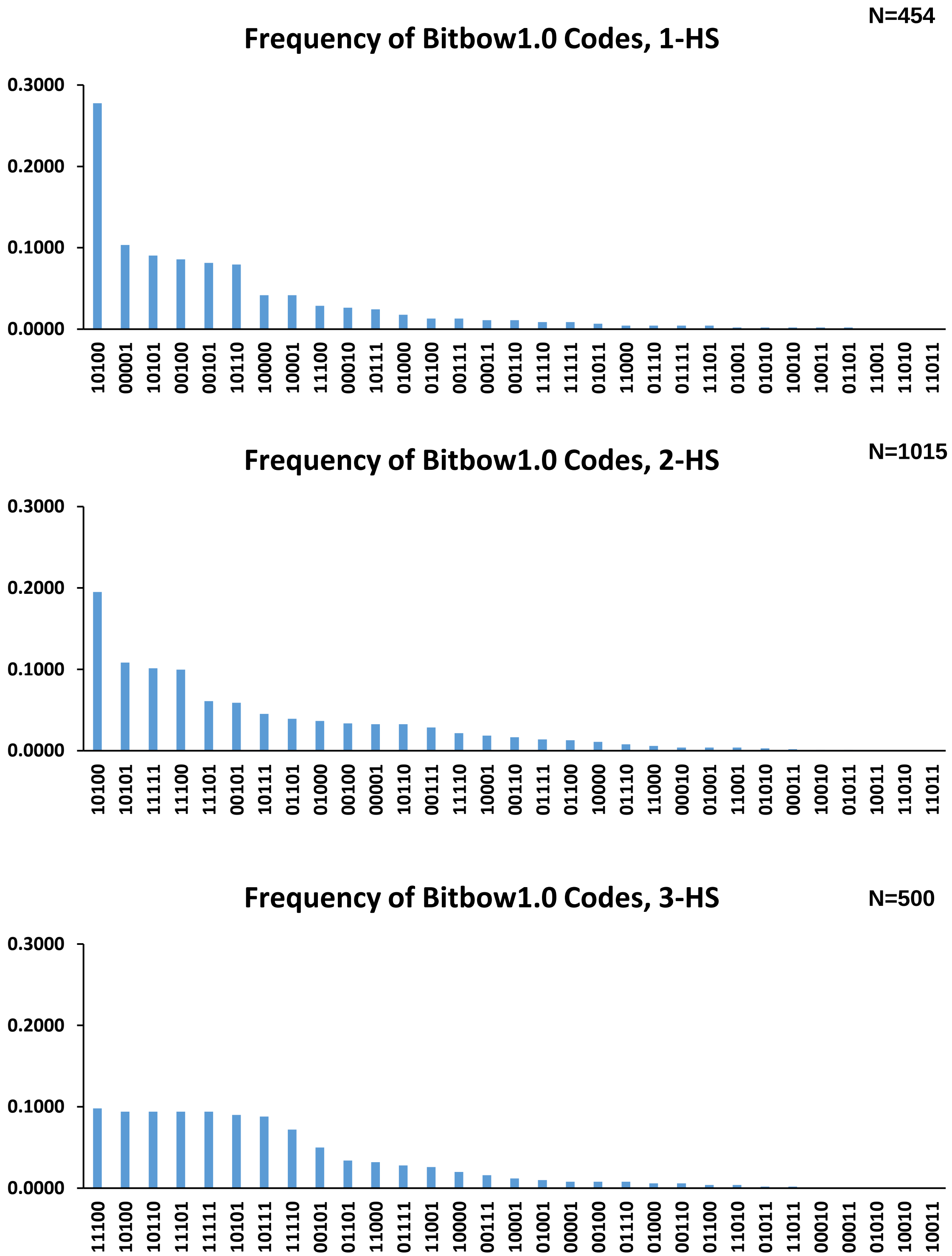

Figure S5. Observed frequency of the 31 mBitbow1.0 codes upon heat-shock induced Flp recombination. Observed frequency of each Bitbow code, when the flies underwent (A) one, (B) two, or (C) three heat shocks.

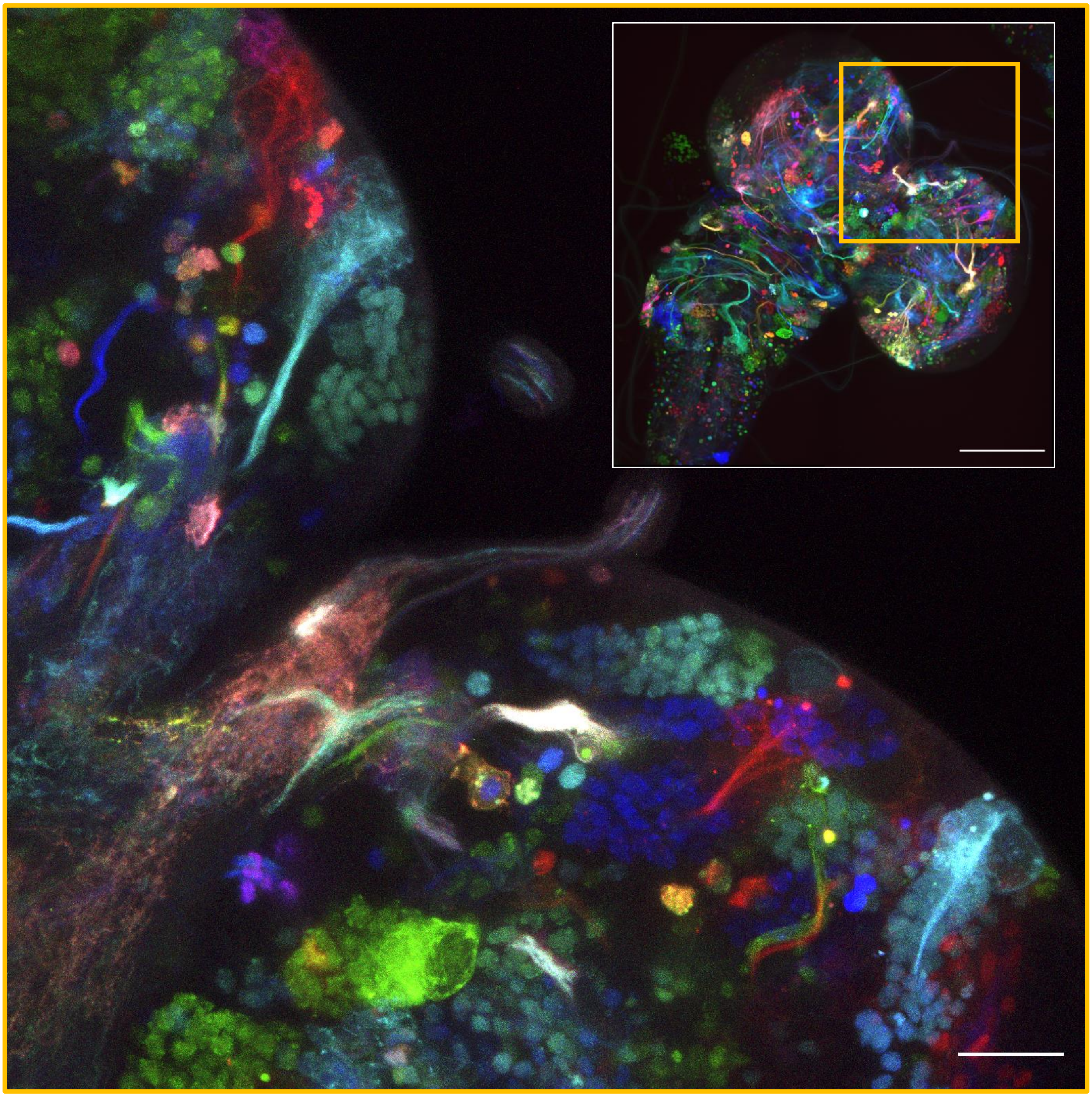

Figure S6. Third-instar larva neuronal clusters labeled by mnBitbow1.0. A hsFlp;;elav-Gal4 driver fly was crossed to the membrane and nucleus dual-targeted mnBitbow1.0 fly, whose offsprings were heat-shock at the 3rd instar larva stage, similar to the protocol shown in **Figure 2C**. The white box shows the maximum intensity projection of the whole brain overview and the yellow boxed region is magnified to show a partial z-plane maximum intensity projection. Scale bars: overview 100μm, magnified 20μm.

A

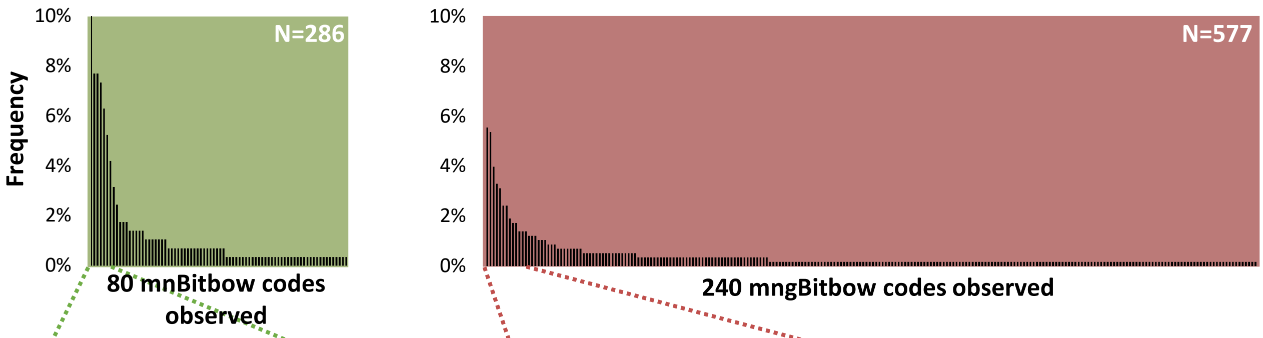

B

Rank	mnBitbow code	Frequency
1	0000000100	10.49%
2	0000000110	7.69%
3	0010000000	7.69%
4	0000001000	7.34%
5	0010000100	6.29%
6	0000001100	5.24%
7	0000010100	4.20%
8	0011000000	3.15%
9	0000100000	2.45%
10	0000010110	1.75%

Rank	mngBitbow code	Frequency
1	000000000000100	5.55%
2	000000000000001	5.37%
3	000000010000000	3.99%
4	001000000000000	3.29%
5	010000000000000	3.12%
6	000000000000110	2.43%
7	001000010000000	2.43%
8	000000010000100	1.91%
9	000000100000000	1.73%
10	001000000000100	1.73%

Figure S7. Statistics of observed mnBitbow and mngBitbow codes. (A) Histograms of the mnBitbow or mngBitbow code frequencies observed from brains sampled in **Fig. 2**. The observed Shannon entropy of the mnBitbow and mngBitbow codes are 5.9 and 8.5, respectively. **(B)** Top 10 most frequently observed mnBitbow or mngBitbow codes. Bits corresponding to mNeonGreen expressions were highlighted in green.

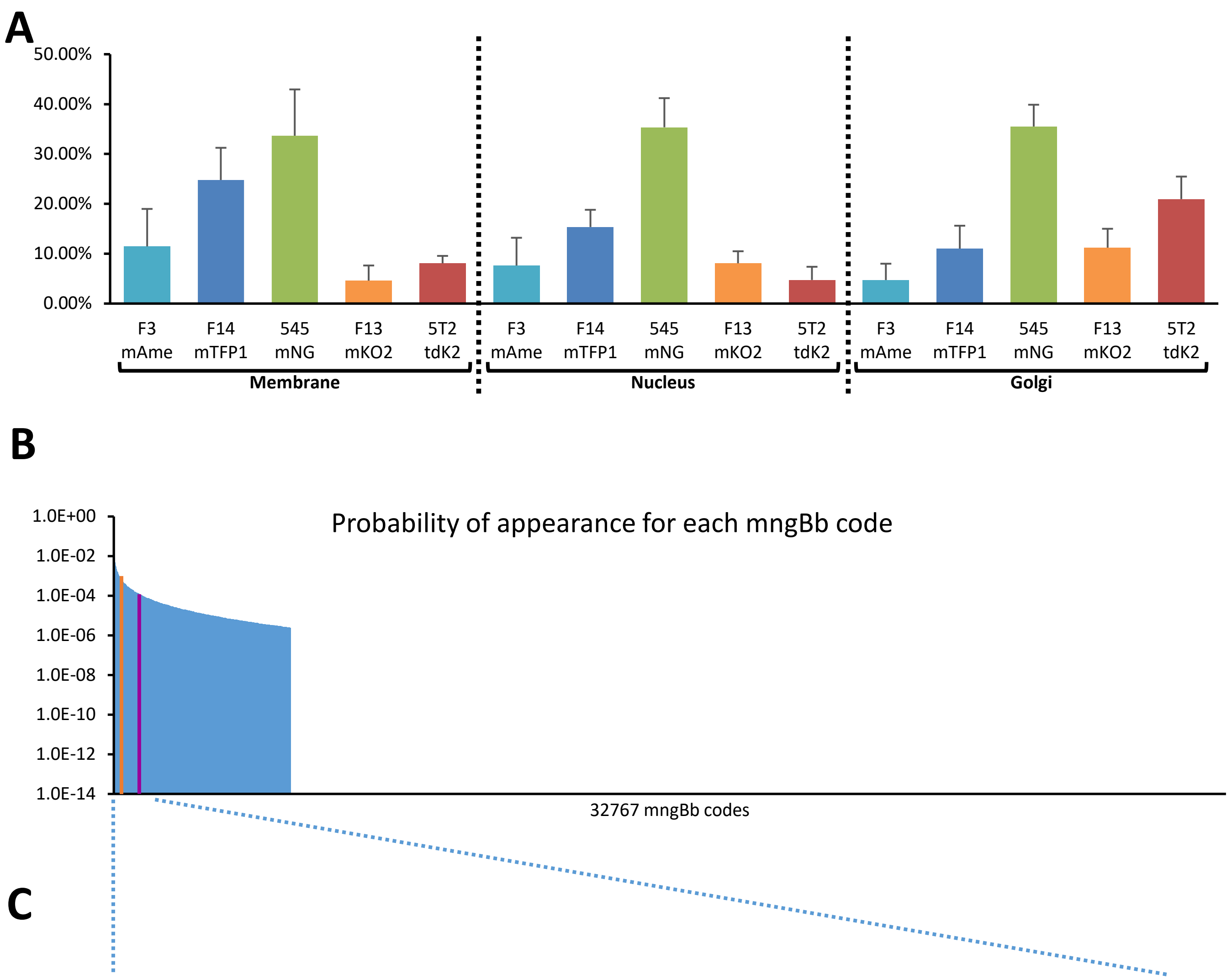

	000000000000100	000000010000000	001000000000000	010000000000000	000000010000100	001000000000100	001000010000000		100000010000100	000000010000110	000000010001100		101010010000100	101000011000100	001000010100010	
p-value	0.0362	0.0360	0.0334	0.0217	0.0198	0.0184	0.0183	...	2.57E-03	2.50E-03	2.46E-03	...	1.15E-04	1.15E-04	1.15E-04	...
rank	32767	32766	32765	32764	32763	32762	32761	...	32701	32700	32699	...	32001	32000	31999	...

Figure S8. Estimation of the experimental frequencies of mngBitbow1.0 codes. (A) Observed 8frequencies of the 15 recombination events in mngBitbow1.0. (N=577 clusters from 6 brains) **(B)** Based on the observed frequencies quantified in (A), we synthesized the probability of occurrence of all 32767 mngBitbow codes. **(C)** High-probability mngBitbow codes and their corresponding p-values are sorted from high probability to low probability. Bits corresponding to mNeonGreen expressions are highlighted in green. Code cutoffs at rank 32700 and 32000 used in **Fig. 2G** and **2J** are highlighted in orange and purple, respectively.

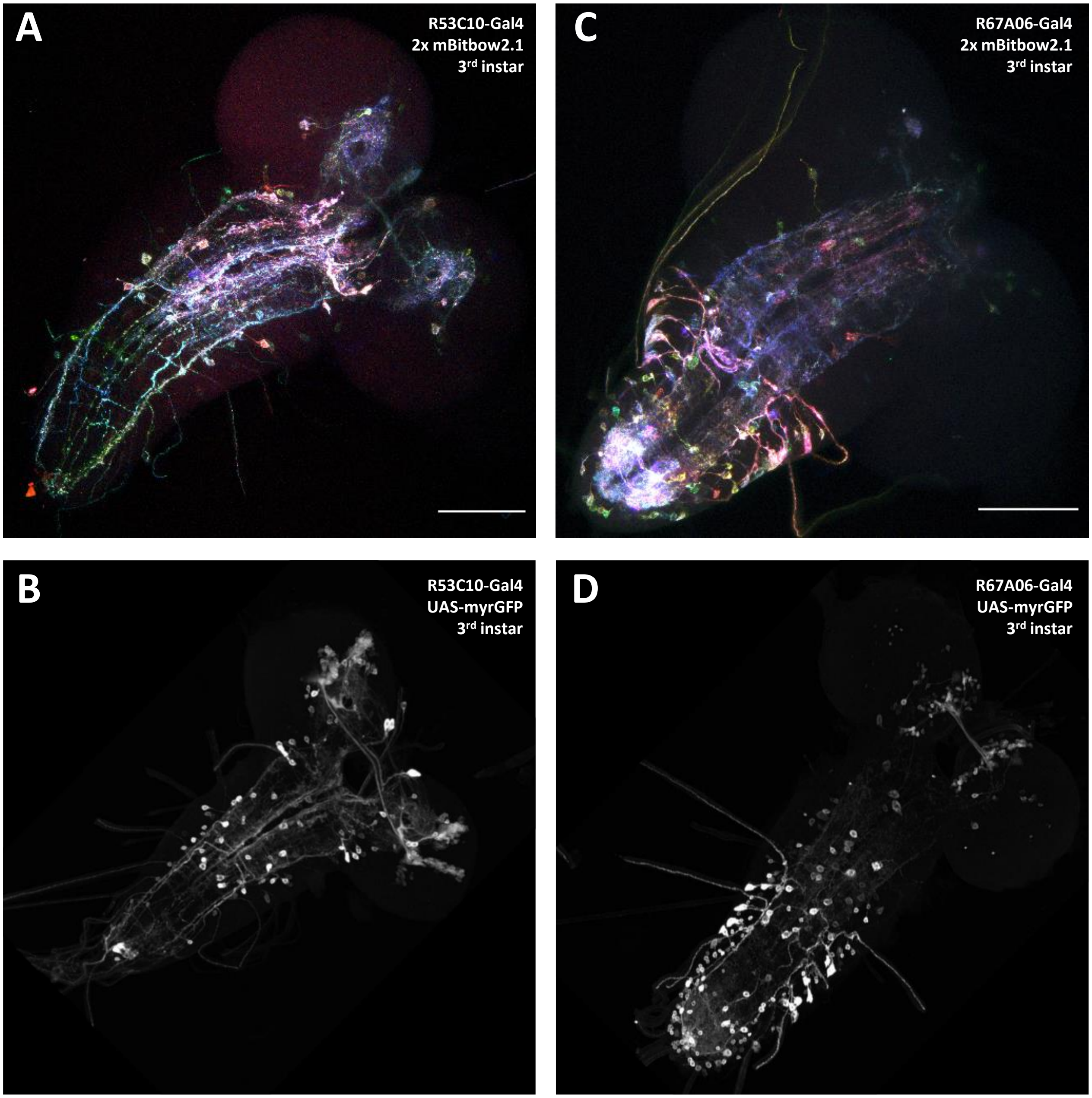

Figure S9. Bitbow2.1 labels different subsets of neurons when directly crossed to specific enhancer-Gal4 drivers. (A, B) 3rd instar neurons labeled in an offspring of the 2x mBitbow2.1 fly or UAS-myrGFP (from Janelia FlyLight collection) crossed to the R53C10-Gal4 fly, respectively. **(C, D)** 3rd instar neurons labeled in an offspring of the 2x mBitbow2.1 fly or UAS-myrGFP (from Janelia FlyLight collection) crossed to the R67A06-Gal4 fly, respectively. Scale bars: 100µm.

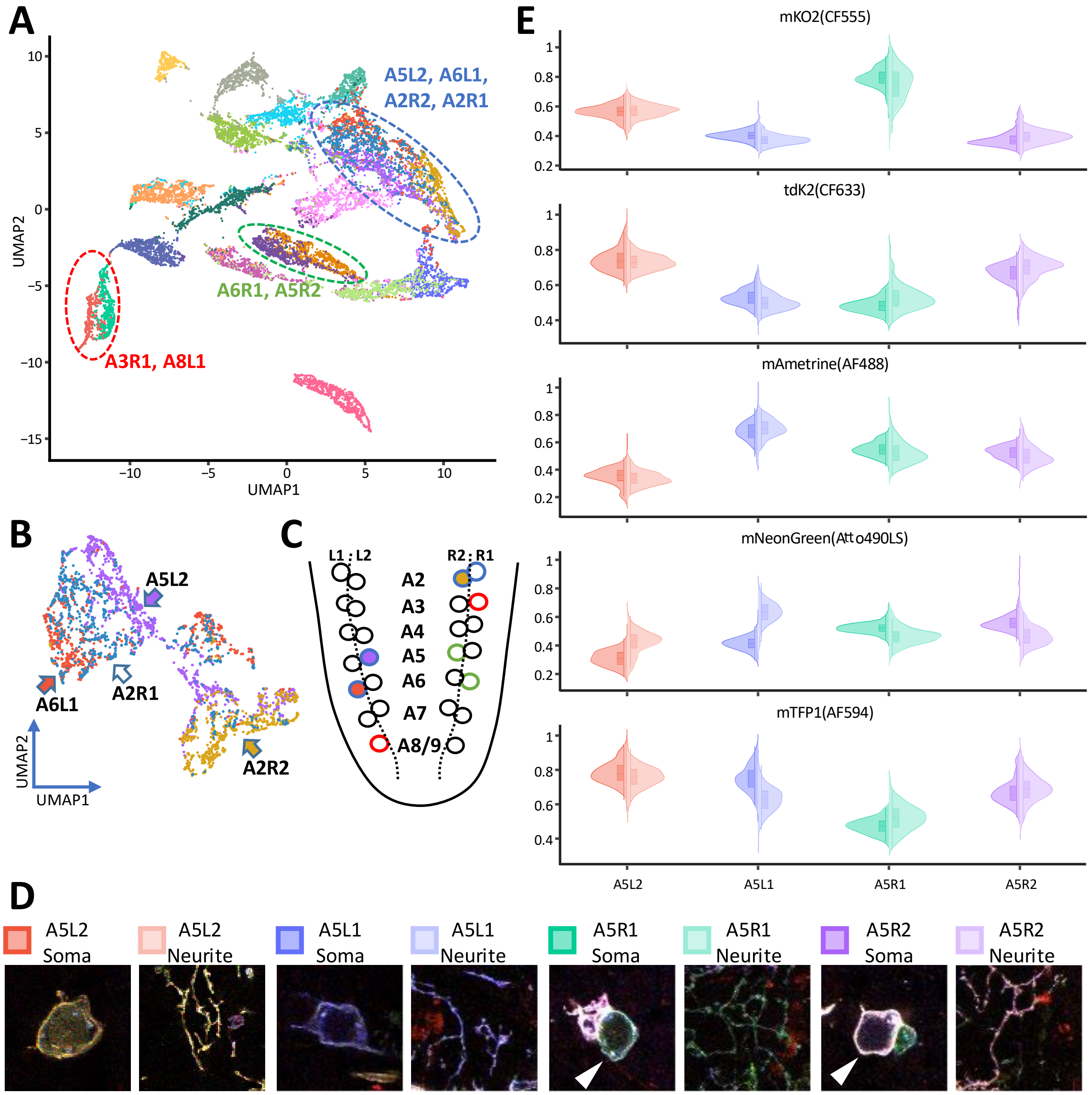

Figure S10. Bitbow color analysis of the traced 21 larval VNC serotonergic neurons. (A) The normalized pixel intensities in each Bitbow channel along the 21 neurons' soma tracing were recorded and projected as a 2D UMAP display. 16 well-separated clusters, representing 16 resolvable Bitbow colors are identified on the UMAP embedding. 3 of the 16 clusters contain pixels sampled from more than one neuron, marked by red, green or blue dashed circles with the neuron names indicated in the same colors. Among these 3 clusters, the red- and green-circled clusters contain 2 neurons each and the two neurons composed consistent subtle spectral differences that were indicated by their tight pixel locations within the same cluster. The blue-circled cluster contains 4 neurons, whose pixel locations are much more intermingled on this UMAP. **(B)** We took those pixels belonging to the blue-circled cluster and remapped them on a higher resolution 2D UMAP display and found that 3 of the 4 neurons can be separated by consistent subtle spectral differences (solid arrows). **(C)** We marked the soma positions of the 8 neurons with less separable Bitbow colors in a

schematic of the VNC serotonergic neurons. The circle outline colors correspond to the 3 dashed color line-circled clusters in (A). For the blue-circled neurons, their filled colors correspond to the filled colors of the arrows in (**B**). **(D)** Example images of the soma and distal neurites of the four A5 neurons displayed in **Fig. 4B**. **(E)** Half-violin plots of the four A5 neurons' normalized color intensities in each Bitbow spectral channel. Left and right halves plot pixels sampled from soma tracings and distal neurite tracings, respectively. Plotting colors correspond to the same square colors in (**D**), which indicate different A5-neurons. Boxes inside the violin plots are 1st quartile, median and 3rd quartile indicators. Y-axis, normalized fluorescence intensity in each channel.

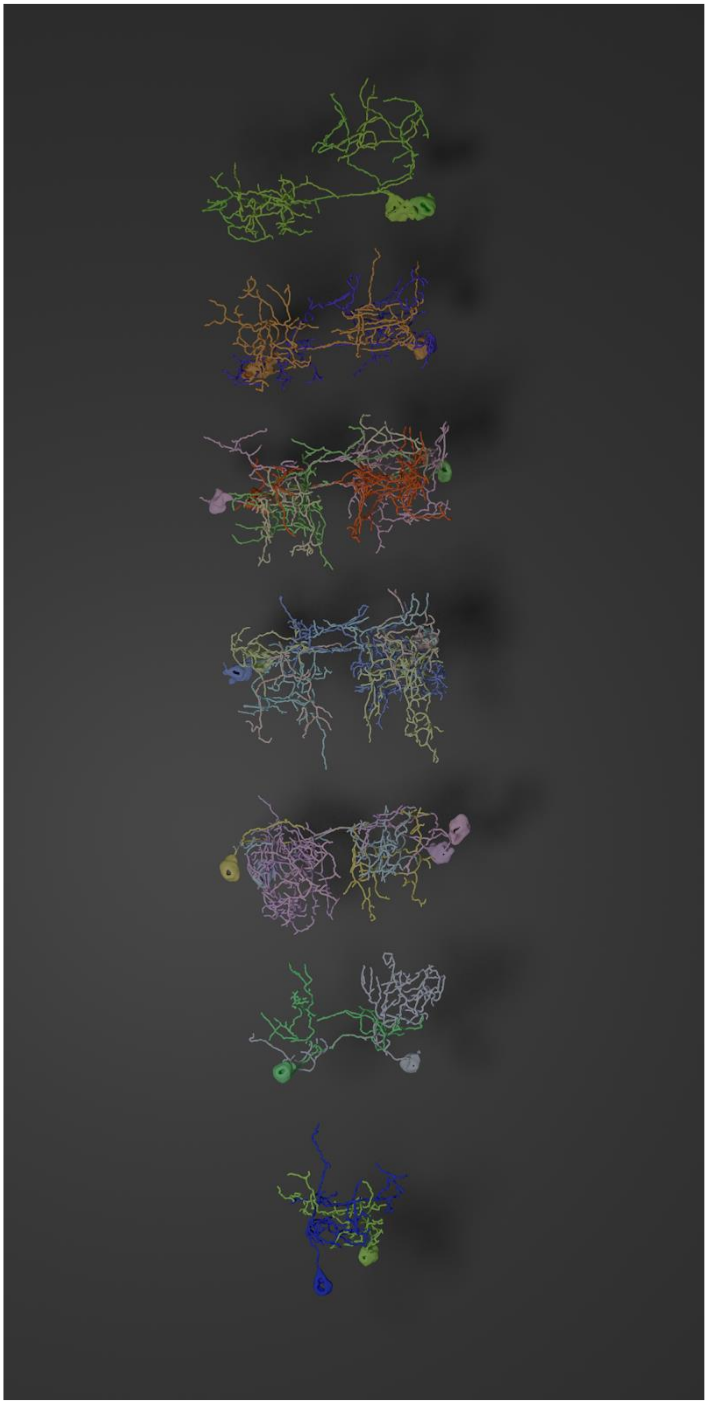

Figure S11. Segmental display of tracings from abdominal serotonergic neurons in a Drosophila larva. Segments were organized from anterior to posterior, as the top neurons belong to segment A2, and the bottom neurons belong to segment A8.

2 Supplementary Movies

Movie S1. Bitbow2 labeled VNC serotonergic neurons traced by nTracer. Three VNC hemi-segments of an offspring of the Bitbow2.1 fly and TRH-Gal4 fly cross are processed by a modified protein-retention Expansion Microscopy protocol and imaged by a confocal microscope. The nTracer results are overlaid on the fluorescence image and 3D rendering of the tracing results is shown to illustrate the relative projection patterns of the serotonergic neurons.

Movie S2. Animations illustrating the imaging, tracing and morphology analysis of a Bitbow2 labeled fly. First, the volumetric fluorescent data is overviewed, followed by the reveal of the nTracer-generated tracings. Finally, all neurons are then separated into manually-assigned morphological groupings, as in **Fig. 4**.
